# Supplementary material for: Randomised controlled trial to establish the clinical and cost-effectiveness of expectant management versus preoperative imaging with magnetic resonance cholangiopancreatography in patients with symptomatic gallbladder disease undergoing laparoscopic cholecystectomy at low or moderate risk of common bile duct stones (The Sunflower Study): a study protocol
Source: BMJ Open. 2021 Jun 29;11(6):e044281. doi: 10.1136/bmjopen-2020-044281 (PMC8245448; doi:10.1136/bmjopen-2020-044281)
Supplement: Supplementary data [file bmjopen-2020-044281supp001.pdf]

**Patient Study ID** ☐

Place Trust logo and

PI details sticker here

**HTA reference:** 16/142/04
**IRAS Project ID:** 242342

# The Sunflower Study

**Testing for bile duct stones before gallbladder surgery**

## Participant Consent Form

***Please ask the patient to complete the following:*** ***Initial***

1. I have read and understood the Patient Information Leaflet (dated ____/____/____, version _____ ). ☐
2. I have had the opportunity to ask questions about the study and received satisfactory answers to my questions. ☐
3. I understand that I am free to withdraw from the study at any time without giving a reason. I understand that a decision to withdraw will not affect my medical care or legal rights. ☐
4. I give permission for information about me to be collected and shared with the Sunflower Study researchers, researchers from the University of Bristol, University Hospitals Bristol NHS Foundation Trust and Leeds Teaching Hospitals NHS Trust. This will include information collected specifically for the study and information collected as part of my routine care. I understand that strict confidentiality will be maintained at all times. ☐
5. I give permission for my date of birth, full name, NHS/CHI number, gender and post code to be sent to <<insert text e.g. NHS Digital, PEDW>> and providers of mortality data. I understand that data held about me on these systems including hospital episodes, diagnostic imaging and mortality data will be sent to the Sunflower Study researchers at University Hospitals Bristol NHS Foundation Trust. ☐
6. I agree to my GP being informed that I have agreed to take part in this study and to them providing relevant information from my records. ☐
7. I understand that my MRCP scan images may be sent to a central study team at Leeds Teaching Hospitals NHS Trust for quality assurance purposes. Scans will be transferred securely using the NHS Image Exchange Portal. I understand that strict confidentiality will be maintained at all times. ☐
8. I agree to take part in this study. ☐

| ***Optional section – if you do not agree to any of the following you can still take part in Sunflower*** | ***Yes*** | ***No*** | ***Initial*** |
|---|---|---|---|
| 9. I agree to my information being stored securely for potential use in future ethically approved research. | ☐ | ☐ | ☐ |

***Patient to tick Yes/No and initial***

________________________ ____________________ ______________
Name of patient Signature Date

________________________ ____________________ ______________
Name of person taking consent Signature Date

*Funded & supported by* **NIHR**

**1 copy for patient; 1 for research team (original); 1 to be kept with hospital notes.**

**The Sunflower Study is funded by the NIHR HTA Programme (project number 16/142/04). The views expressed are those of the author(s) and not necessarily those of the NHS, the NIHR or the Department of Health.**
