# Supplementary material for: Randomised controlled trial to establish the clinical and cost-effectiveness of expectant management versus preoperative imaging with magnetic resonance cholangiopancreatography in patients with symptomatic gallbladder disease undergoing laparoscopic cholecystectomy at low or moderate risk of common bile duct stones (The Sunflower Study): a study protocol
Source: BMJ Open. 2021 Jun 29;11(6):e044281. doi: 10.1136/bmjopen-2020-044281 (PMC8245448; doi:10.1136/bmjopen-2020-044281)
Supplement: Author's manuscript [file bmjopen-2020-044281.draft_revisions.pdf]

BMJ Open is committed to open peer review. As part of this commitment we make the peer review history of every article we publish publicly available.

When an article is published we post the peer reviewers' comments and the authors' responses online. We also post the versions of the paper that were used during peer review. These are the versions that the peer review comments apply to.

The versions of the paper that follow are the versions that were submitted during the peer review process. They are not the versions of record or the final published versions. They should not be cited or distributed as the published version of this manuscript.

BMJ Open is an open access journal and the full, final, typeset and author-corrected version of record of the manuscript is available on our site with no access controls, subscription charges or pay-per-view fees (http://bmjopen.bmj.com).

If you have any questions on BMJ Open's open peer review process please email info.bmjopen@bmj.com

BMJ Open

Study protocol for a randomised controlled trial to establish the clinical and cost effectiveness of expectant management versus pre-operative imaging with magnetic resonance cholangiopancreatography in patients with symptomatic gallbladder disease undergoing laparoscopic cholecystectomy at low or moderate risk of common bile duct stones (The Sunflower Study)

Journal:	*BMJ Open*
Manuscript ID	bmjopen-2020-044281
Article Type:	Protocol
Date Submitted by the Author:	28-Aug-2020

Complete List of Authors:	Clout, Madeleine; University of Bristol Faculty of Medical and Veterinary Sciences, Clinical Trials and Evaluation Unit<br>Blazeby, Jane; University of Bristol, Department of Social Medicine<br>Rogers, Chris; university of bristol, Clinical Science South Bristol<br>Reeves, Barnaby; University of Bristol, Bristol Heart Institute<br>Lazaroo, Michelle; University of Bristol Faculty of Medical and Veterinary Sciences, Clinical Trials and Evaluation Unit<br>Avery, Kerry; University of Bristol, School of Social and Community Medicine<br>Blencowe, Natalie; University of Bristol, Centre for Surgical Research, School of Social and Community Medicine<br>Vohra, Ravi; Nottingham City Hospital<br>Jennings, Neil; Sunderland Royal Hospital<br>Hollingworth, William; University of Bristol, School of Social and Community Medicine<br>Thorn, Joanna; University of Bristol, School of Social and Community Medicine<br>Jepson, Marcus; University of Bristol School of Social and Community Medicine,<br>Collingwood, Jane; University of Bristol, School of Population Health Sciences<br>Guthrie, Ashley; Leeds Teaching Hospitals NHS Trust<br>Booth, Elizabeth; NHS Coventry and Rugby Clinical Commissioning Group<br>Pathak, Samir; University of Bristol, Centre for Surgical Research, Population Health Sciences<br>Beckingham, Ian; Nottingham University Hospitals NHS Trust<br>Culliford, Lucy; University of Bristol, Bristol Heart Institute,<br>Griffiths, Ewen; University Hospitals Birmingham NHS Foundation Trust, Department of Upper GI Surgery<br>Albazaz, Raneem; Leeds Teaching Hospitals NHS Trust<br>Toogood, Giles; Leeds Teaching Hospitals NHS Trust

Keywords:	Magnetic resonance imaging < RADIOLOGY & IMAGING, Endoscopy < GASTROENTEROLOGY, SURGERY, GASTROENTEROLOGY, NEPHROLOGY,

	Clinical trials < THERAPEUTICS

SCHOLARONE™
Manuscripts

I, the Submitting Author has the right to grant and does grant on behalf of all authors of the Work (as defined in the below author licence), an exclusive licence and/or a non-exclusive licence for contributions from authors who are: i) UK Crown employees; ii) where BMJ has agreed a CC-BY licence shall apply, and/or iii) in accordance with the terms applicable for US Federal Government officers or employees acting as part of their official duties; on a worldwide, perpetual, irrevocable, royalty-free basis to BMJ Publishing Group Ltd ("BMJ") its licensees and where the relevant Journal is co-owned by BMJ to the co-owners of the Journal, to publish the Work in this journal and any other BMJ products and to exploit all rights, as set out in our licence.

The Submitting Author accepts and understands that any supply made under these terms is made by BMJ to the Submitting Author unless you are acting as an employee on behalf of your employer or a postgraduate student of an affiliated institution which is paying any applicable article publishing charge ("APC") for Open Access articles. Where the Submitting Author wishes to make the Work available on an Open Access basis (and intends to pay the relevant APC), the terms of reuse of such Open Access shall be governed by a Creative Commons licence – details of these licences and which Creative Commons licence will apply to this Work are set out in our licence referred to above.

Other than as permitted in any relevant BMJ Author's Self Archiving Policies, I confirm this Work has not been accepted for publication elsewhere, is not being considered for publication elsewhere and does not duplicate material already published. I confirm all authors consent to publication of this Work and authorise the granting of this licence.

For peer review only

Study protocol for a randomised controlled trial to establish the clinical and cost effectiveness of expectant management versus pre-operative imaging with magnetic resonance cholangiopancreatography in patients with symptomatic gallbladder disease undergoing laparoscopic cholecystectomy at low or moderate risk of common bile duct stones (The Sunflower Study)

On behalf of the Sunflower Study Executive Group*

*Authors for 'Sunflower Study Executive Group':

1. Madeleine Clout, Clinical Trials and Evaluation Unit, Bristol Trials Centre, University of Bristol, Bristol Royal Infirmary, Zone A Level 7, Bristol, BS2 8HW, UK, madeleine.clout@bristol.ac.uk
2. Jane Blazeby, NIHR Bristol Biomedical Research Centre, Centre for Surgical Research, Bristol Medical School, Population Health Sciences, University of Bristol, Office 4.05 Canynge Hall, 39 Whatley Road, Bristol, BS8 2PS, UK, j.m.blazeby@bristol.ac.uk
3. Chris Rogers, Clinical Trials and Evaluation Unit, Bristol Trials Centre, University of Bristol, Bristol Royal Infirmary, Zone A Level 7, Bristol, BS2 8HW, UK, chris.rogers@bristol.ac.uk
4. Barnaby C Reeves, Clinical Trials and Evaluation Unit, Bristol Trials Centre, University of Bristol, Bristol Royal Infirmary, Zone A Level 7, Bristol, BS2 8HW, UK, barney.reeves@bristol.ac.uk
5. Michelle Lazaroo, Clinical Trials and Evaluation Unit, Bristol Trials Centre, University of Bristol, Bristol Royal Infirmary, Zone A Level 7, Bristol, BS2 8HW, UK, michelle.lazaroo@bristol.ac.uk
6. Kerry Avery, NIHR Bristol Biomedical Research Centre and Centre for Surgical Research Bristol Medical School, Population Health Sciences, University of Bristol, 39 Whatley Road, Bristol, BS8 2PS, UK, Kerry.Avery@bristol.ac.uk

For peer review only

7. Natalie Blencowe, NIHR Bristol Biomedical Research Centre and Centre for Surgical Research, Population Health Sciences, Bristol Medical School, University of Bristol, 39 Whatley Road, Bristol, BS8 2PS, UK, Natalie.Blencowe@bristol.ac.uk
8. Ravinder Vohra, Trent Oesophago-Gastric Unit, Nottingham University Hospitals NHS Trust, City Hospital, Hucknall Rd, Nottingham, NG5 1PB, UK, ravinder.vohra@nuh.nhs.com
9. Neil Jennings, Sunderland Royal Hospital, Kayll Road, Sunderland, Tyne & Wear, SR4 7TP, UK, Neil.Jennings@chsft.nhs.uk
10. William Hollingworth, Bristol Medical School, Population Health Sciences, University of Bristol, Office 2.08, 1-5 Whiteladies Road, Bristol, BS8 1NU, UK, william.hollingworth@bristol.ac.uk
11. Joanna Thorn, Bristol Medical School, Population Health Sciences, University of Bristol, Office 2.12, 1-5 Whiteladies Road, Bristol, BS8 1NU, UK, joanna.thorn@bristol.ac.uk
12. Marcus Jepson, Bristol Medical School, Population Health Sciences, University of Bristol, Office 4.03 Canynge Hall, 39 Whatley Road, Bristol, BS8 2PS, UK, marcus.jepson@bristol.ac.uk
13. Jane Collingwood, Bristol Medical School, Population Health Sciences, University of Bristol, Office 2.01 Canynge Hall, 39 Whatley Road, Bristol, BS8 2PS, UK, jane.collingwood@bristol.ac.uk
14. Ashley Guthrie, Leeds Teaching Hospitals NHS Trust, St James's University Hospital, Beckett Street, Leeds, LS9 7TF, UK, ashley.guthrie@nhs.net
15. Elizabeth Booth, NHS Coventry and Rugby Clinical Commissioning Group, Parkside House, Quinton Road, Coventry, CV1 2NJ, UK, elizabeth.booth@coventryrugbyccg.nhs.uk
16. Samir Pathak, Centre for Surgical Research and Centre for Surgical Research, Population Health Sciences, Bristol Medical School, University of Bristol, 39 Whatley Road, Bristol, BS8 2PS, UK, drsamirpathak@gmail.com

For peer review only

For peer review only - http://bmjopen.bmj.com/site/about/guidelines.xhtml

17. Ian Beckingham, Nottingham University Hospitals NHS Trust, Hucknall Road, Nottingham, NG5 1PB, UK, ian.beckingham@nuh.nhs.uk
18. Lucy Culliford, Clinical Trials & Evaluation Unit, Bristol Trials Centre, University of Bristol, Bristol Royal Infirmary, Zone A Level 7, Bristol, BS2 8HW, UK, lucy.culliford@bristol.ac.uk
19. Ewen A Griffiths, University Hospitals Birmingham NHS Foundation Trust, Queen Elizabeth Hospital Birmingham, Mindelsohn Way, Edgbaston, Birmingham, B15 2GW, UK, Ewen.Griffiths@uhb.nhs.uk
20. Raneem Albazaz, Leeds Teaching Hospitals NHS Trust, St James's University Hospital, Beckett Street, Leeds, LS9 7TF, UK, r.albazaz@nhs.net
21. Giles Toogood, Department of Hepatobiliary and Transplantation Surgery, Leeds Teaching Hospitals NHS Trust, St James's University Hospital, Beckett Street, Leeds, LS9 7TF, UK, giles.toogood@nhs.net

Corresponding author

Miss Madeleine Clout

madeleine.clout@bristol.ac.uk

Clinical Trials and Evaluation Unit

Bristol Trials Centre

University of Bristol

Bristol Royal Infirmary, Zone A Level 7

Bristol

BS2 8HW

0117 342 2526 / 07929 771395

For peer review only

For peer review only - http://bmjopen.bmj.com/site/about/guidelines.xhtml

Word count: 3994

Keywords: Gallstones, gallbladder disease, laparoscopic cholecystectomy, magnetic resonance cholangiopancreatography, randomised controlled trial, common bile duct, endoscopic retrograde cholangiopancreatography

For peer review only

For peer review only - http://bmjopen.bmj.com/site/about/guidelines.xhtml

ABSTRACT:

Introduction

Surgery to remove the gallbladder (laparoscopic cholecystectomy (LC)) is the standard treatment for symptomatic gallbladder disease. One potential complication of gallbladder disease is that gallstones can pass into the common bile duct (CBD) where they may remain dormant, pass spontaneously into the bowel or cause problems such as obstructive jaundice or pancreatitis. Patients requiring LC are assessed pre-operatively for their risk of CBD stones using liver function tests and imaging. If the risk is high, guidelines recommend further investigation and treatment. Further investigation of patients at low or moderate risk of CBD stones is not standardised, and the practice of imaging the CBD using magnetic resonance cholangiopancreatography (MRCP) in these patients varies across the United Kingdom (UK). The consequences of these decisions may lead to over or under treatment of patients.

Methods and analysis

We are conducting a UK multicentre, pragmatic, open, randomised controlled trial with internal pilot phase to compare the effectiveness and cost-effectiveness of pre-operative imaging with MRCP versus expectant management (i.e. no pre-operative imaging) in adult patients with symptomatic gallbladder disease undergoing urgent or elective LC who are at low or moderate risk of CBD stones. We aim to recruit 13,680 patients over 48 months. The primary outcome is any hospital admission within 18 months of randomisation for a complication of gallstones. This includes complications of endoscopic retrograde cholangiopancreatography for the treatment of gallstones, and complications of LC. This will be determined using routine data sources, e.g. NHS Digital Hospital Episode Statistics for participants in England. Secondary outcomes include cost-effectiveness and patient-reported quality of life, with participants followed up for a median of 18 months.

Ethics and dissemination

For peer review only

This study received approval from Yorkshire & The Humber – South Yorkshire Research Ethics Committee. Results will be submitted for publication in a peer-reviewed journal.

Registration details: ISRCTN10378861

For peer review only

For peer review only - http://bmjopen.bmj.com/site/about/guidelines.xhtml

ARTICLE SUMMARY

Strengths and limitations of this study

- This is a randomised controlled trial with randomisation taking place using a secure internet-based system to ensure allocation concealment, which will ensure that patients allocated to each group have comparable baseline characteristics.
- The study includes an internal pilot phase with progression to the main study dependent on a number of criteria, to ensure that the study will only proceed if it is viable to do so.
- Participants, clinicians and other hospital staff caring for participants will not be blind to the study allocation.
- The criteria used to define low and moderate risk of common bile duct stones have been identified by clinicians within the study team, as there is no universal agreed definition of these terms.
- The study primary outcome will be derived from routine data sources, which should ensure high levels of data completeness but is reliant on timely approval of numerous applications to holders of routine data.

For peer review only

INTRODUCTION

Surgery to remove the gallbladder, known as laparoscopic cholecystectomy (LC), is the standard treatment for symptomatic gallbladder disease. LC is one of the most common operations undertaken globally and around 70,000 LC operations are performed annually in England.[1]

One potential complication of gallbladder disease is that gallstones may pass from the gallbladder into the common bile duct (CBD). Once in the CBD, they may remain dormant, pass spontaneously into the bowel or cause problems such as obstructive jaundice, cholangitis or pancreatitis. If CBD stones are found, current guidelines recommend removal either before or during LC. Therefore, patients requiring LC are assessed pre-operatively for their risk of CBD stones using a combination of liver function tests (LFTs) and imaging. If the risk is high, guidelines recommend further investigation and treatment.[2-6] Further investigation of patients at moderate or low risk of CBD stones is not standardised and practice across the United Kingdom (UK) varies. Fewer than 10% of these patients actually have CBD stones.[2-4, 6, 7] Consequently, surgeons make different decisions about investigation of the CBD; some surgeons choose to perform diagnostic imaging tests for these patients, and others do not. This variation likely results in over or under treatment, with significant risks to the patient in terms of morbidity, Health Related Quality of Life (HRQoL) and costs to the health service.

For peer review only

A UK-wide audit found that a third of patients undergoing LC, irrespective of risk of CBD stones, had diagnostic imaging to test for CBD stones.[7] This is most frequently performed with a magnetic resonance cholangiopancreatography (MRCP) before admission for LC, however some surgeons instead use intra-operative cholangiogram and other intra-operative ultrasound examinations of the CBD. MRCP produces detailed images of the biliary and pancreatic ducts, involves a 1-hour hospital visit and costs the National Health Service (NHS) about £365. MRCP can identify CBD stones but may delay LC, which can have a detrimental effect on HRQoL and can lead to increased problems with gallstones or a more complex LC.[8] If a CBD stone is identified during MRCP, extraction is most

For peer review only - http://bmjopen.bmj.com/site/about/guidelines.xhtml

frequently performed before LC with an endoscopic retrograde cholangiopancreatography (ERCP). The risks and mortality associated with ERCP are significant,[9] as is the cost to the NHS (about £1,600). Not having MRCP avoids these risks but can lead to CBD stones remaining after surgery, which may also cause complications. Research is needed to establish whether it is appropriate for patients at low and moderate risk of CBD stones to proceed straight to LC without imaging the CBD with MRCP.

METHODS AND ANALYSIS

Aims and Objectives

The Sunflower study aims to compare the effectiveness and cost-effectiveness of pre-operative imaging with MRCP versus no MRCP (hereafter called expectant management (EM)) in patients with symptomatic gallbladder disease undergoing LC who are at low or moderate risk of CBD stones. The study will test the hypothesis that EM is not inferior to MRCP with respect to hospitalisation for treatment for a complication of gallstones up to 18 months after randomisation.

The objectives are to estimate the:

1. Difference between groups in the proportion of participants requiring a hospital admission for treatment of a complication of gallstones in the gallbladder or CBD.
2. Difference between groups with respect to a range of secondary outcomes, including patient-reported HRQoL, symptoms related to complications of gallstones in the gallbladder or CBD, and symptoms related to complications associated with LC and, where applicable, ERCP.
3. Cost-effectiveness of MRCP compared with EM.

For peer review only - http://bmjopen.bmj.com/site/about/guidelines.xhtml

Study Design

The Sunflower study is a multi-centre, pragmatic, open, randomised controlled trial with an internal pilot. The study has an embedded Qualitative Research Integrated within Trials Recruitment Intervention (QRI) to optimise informed consent and recruitment.[10]

There are two phases to the study:

Phase 1 (internal pilot): Set-up and recruit from at least 36 centres by month 16 of recruitment, with integrated QRI to optimise recruitment.

Phase 2: Increase the number of centres to at least 50 and continue recruitment using the methods established in Phase 1, along with integrated QRI to optimise recruitment and promote adherence in new centres.

Progression to phase 2 is conditional on the study demonstrating to the funder that: a) at least 30 centres are open and recruiting; b) at least 1,750 participants have been randomised; c) at least 90% of participants have followed their allocated pathway; and d) the primary outcome can be identified reliably from routine data.

Setting

Recruitment will take place at a minimum of 50 NHS hospitals across the UK with a recruitment target of 13,680 participants over a 48-month period (

Figure **1**).

Study Population

The target population is adults (18 years or older) with symptomatic gallbladder disease, scheduled and fit for LC as an elective or urgent procedure, who are at low or moderate risk of CBD stones. Low

For peer review only - http://bmjopen.bmj.com/site/about/guidelines.xhtml

or moderate risk is defined as CBD diameter ≤8mm on ultrasound scan (USS), and bilirubin ≤50umol/l, and alanine transferase (ALT) less than three times the upper limit of normal (≤3xULN) and/or alkaline phosphatase (ALP) ≤3xULN. If the CBD cannot be visualised on USS, the patient may be recruited if no intrahepatic duct dilatation is reported.

Patients are excluded if they are unable to undergo MRCP, have evidence of empyema or perforated gallbladder requiring urgent intervention, have had a previous gastric bypass, have had a previous MRCP or endoscopic USS within the last 3 months, have had any previous ERCP or have haemolytic disease. Patients must be willing to participate in follow up and be able to provide informed consent.

Figure 1: The study schema

Primary Outcome

The primary outcome is any of the following:

i. Any hospital admission within 18 months of randomisation for treatment of a complication of gallstones whether in the CBD or gallbladder;
ii. Complications during the admission for LC for the treatment for gallstones or any readmission for complications of the LC leading to a hospital day of >2 days. Complications will include, but not be limited to, return to theatre post LC for any cause, percutaneous radiological drainage and ERCP for non-diagnostic reasons (e.g. for a bile leak). It does not include a diagnostic ERCP performed following an MRCP where CBD stones were identified;
iii. Complications during any ERCP for the treatment for gallstones. Complications will include, but are not limited to, blood transfusion post ERCP, percutaneous radiological drainage, treatment of a perforation occurring during ERCP, acute pancreatitis and other complications leading to a hospital stay of > 2 days.

For peer review only

For peer review only - http://bmjopen.bmj.com/site/about/guidelines.xhtml

This outcome will be ascertained by linking the study data with routinely-collected Hospital Episode Statistics (HES) and mortality data from NHS Digital for participants recruited in England, and the equivalent data sources for participants recruited in Scotland, Wales and Northern Ireland. Routine data sources contain information on care provided to all patients treated in UK NHS hospitals.[11] The final specification of qualifying events for identifying the primary outcome from these routine sources will be developed and validated during phase 1 of the study, through comparison of routine data with events identified from review of the medical records at 90 days post-LC.

Secondary Outcomes

Patient-reported HRQoL will be measured using the EQ-5D-5L questionnaire.[12] To minimise the overall study burden, about 20% of participants will be sampled and asked to complete questionnaires at multiple timepoints (Table 1). A questionnaire pack will be provided containing the validated EQ-5D-5L questionnaire plus questions about the impact of abdominal pain on work, productivity and primary healthcare use.

For peer review only - http://bmjopen.bmj.com/site/about/guidelines.xhtml

For peer review only

Data item	Pre-Randomisation	Pre-Surgery	Hospital admission for LC	90 days post LC	3 -months[a]	6-months[a]	12-months[a]	18-months[a]
Eligibility	✓							
Written informed consent	✓							
Medical history	✓							
EQ-5D-5L, productivity & primary care use questionnaire (20% sample)	✓		✓[c]		✓	✓	✓	✓
MRCP, ERCP and intraoperative imaging details, if applicable		✓						
Operative and post-operative details			✓					
Items in the symptomatic gallstone core outcome set				✓				
Safety data				✓				
Study consultations audio recorded for QRI [b]	✓							

Table 1: Data collection time points

For peer review only

Other secondary outcomes include items that cover the domains in the core outcome set for patients with symptomatic gallstones, and hospital resource use in the 18 months after randomisation collected from routine data sources.[13, 14]

For peer review only - http://bmjopen.bmj.com/site/about/guidelines.xhtml

Sample Size

The sample size of 13,680 has been chosen to test the hypothesis that EM is non-inferior to MRCP with respect to the primary outcome. In estimating the sample size, we have considered the proportion of patients that would be expected to experience the primary outcome, as identified in the CholeS audit (5% to 10%) and through exploration of HES data.[7] This includes patients at high risk of CBD stones. The consensus amongst clinicians on the study team was that the non-inferiority margin should be set at 1.5%, i.e. that the risk of the primary outcome with EM should not exceed 8.5% assuming a risk of 7% after MRCP. A study of 13,680 participants will provide 90% power to test the non-inferiority hypothesis for a 7% event risk with MRCP and 80% power to test the non-inferiority hypothesis for a higher event risk of 8.5%., assuming 2.5% one-sided statistical significance and a 1:2 allocation ratio (MRCP:EM). The 20% sample of participants providing HRQoL data will be stratified by centre. This sample of 2,736 participants will have >90% power to detect a small effect size of 0.12 standard deviations between groups on the EQ-5D-5L questionnaire.

Recruitment

Recruiting centres will screen LC waiting lists and gallbladder clinic lists for potentially eligible patients. Potential participants will also be identified from urgent inpatient admissions and ward handovers. Eligible patients will be provided with study information and will have the opportunity to ask questions before being approached for their informed consent. Patients who feel they have had insufficient time to consider the study will be invited to take home a consent form and questionnaire pack (where applicable), and to complete and return them by post. Details of all patients approached for the study and reasons for non-participation will be documented.

For peer review only - http://bmjopen.bmj.com/site/about/guidelines.xhtml

266 **Randomisation**

267 Randomisation will be performed by an authorised member of the local research team using a secure internet-based system to ensure allocation concealment. The allocation will be computer generated and stratified by centre.

270 Participants will be randomised to MRCP or EM in a 1:2 ratio. This ratio has been selected to reflect current levels of MRCP provision (13-26% of patients at low or moderate risk of CBD stones currently undergo MRCP).[7]

274 **Interventions**

275 The study interventions are pre-operative MRCP and EM.

276 Participants allocated to the MRCP group will have an MRCP before the date of their listed LC. The study will make no changes to the usual hospital radiology protocols used for MRCP. If a CBD stone is found on MRCP, surgeons can manage extraction according to their usual practice. If an ERCP is requested, it will be performed as a separate inpatient procedure before admission for LC.

280 Participants allocated to EM will be listed for LC without any pre-operative imaging. If imaging is carried out, e.g. due to a change in a participant's clinical circumstances after randomisation, this will be documented as a crossover.

283 In both groups, intraoperative imaging will not be permitted unless there is an anatomical reason to do so, or if a CBD stone is found on MRCP. Any deviations from the protocol will be recorded and monitored.

For peer review only - http://bmjopen.bmj.com/site/about/guidelines.xhtml

Data collection

Data will be collected by the local study team at baseline, before, during and after LC, and at hospital discharge (Table 1). Data collection will be from hospital records, including operative notes, and will include information on any pre-LC MRCP and ERCP, and any intra-operative imaging. Data will either be collected onto a paper case report form and transcribed into a secure electronic study database or entered directly into the database. During phase 1, safety data will also be collected from hospital records 90 days post-LC by the local study team.

Linkage to routine data

With consent, participant identifiers will be sent to NHS Digital, and equivalent organisations for participants recruited outside of England. These organisations will match and extract pseudonymised data for these participants for all episodes of hospital care for a minimum of 18 months from the point of randomisation, and for a period of 12 months prior to randomisation. The data will include inpatient and critical care episodes, accident and emergency department attendances, out-patient appointments, diagnostic imaging investigations and mortality.

MRCP Quality Assurance

To address the challenge of MRCP technique varying across participating centres, images and reports for a random 10% sample of participants who receive an MRCP will be transferred for independent quality assurance review by consultant radiologists. The MRCP examinations will be assessed for the type of sequences performed, the diagnostic quality of the images and level of concordance with the hospital reports. This data will provide an overview of current NHS practice and overall quality of MRCP examinations, as well as characterising variation in MRCP technique in study centres.

For peer review only - http://bmjopen.bmj.com/site/about/guidelines.xhtml

For peer review only

Statistical analysis

The study will be analysed on an intention-to-treat (ITT) basis, i.e. outcomes will be analysed according to the allocated treatment pathway irrespective of future management and events, and every effort will be made to include all randomised participants. Non-adherence to the treatment allocation will be documented. The primary analysis will follow the Consolidated Standards of Reporting Trials guidelines for a non-inferiority study.[15] An analysis according to treatment pathway followed will also be performed for the primary outcome. As recommended, both analyses will be considered when assessing whether the hypothesis is met.[16] The primary outcome will be compared using survival methods to allow for censoring. For participants without a qualifying primary outcome event, they will be censored at the time the dataset was compiled. If more than one qualifying event occurs (e.g. two hospital admissions for gallstone-related complications), time to the first event will be used. The frequency of each element of the primary outcome and reasons for admission, where applicable, will be described. Secondary outcomes will be compared using a mixed model for continuous outcomes or a generalised linear regression model for binary outcomes, adjusted for pre-randomisation measures when available. For secondary outcomes measured at multiple time points, changes in treatment effect with time since randomisation will be assessed by adding a treatment by time interaction to the model and comparing models using a likelihood ratio test. Model fit will be assessed and alternative models and/or transformations (e.g. to induce normality) will be explored where appropriate. Sensitivity analyses using multiple imputation for missing data will be explored. Analyses will be adjusted for centre and treatment differences will be reported with 95% confidence intervals.

A detailed analysis plan will be prepared. There is no intention to compare any outcomes between groups at the end of phase 1; only descriptive statistics about eligibility, recruitment and adherence will be summarised at this time to determine whether the study satisfies the progression criteria.

Exploratory analyses will include:

For peer review only - http://bmjopen.bmj.com/site/about/guidelines.xhtml

i. Relationship between number and size of stones seen on MRCP and patient outcome (cohort undergoing MRCP only);
ii. Relationship between stones removed under ERCP or not (e.g. 'necessary' vs. 'unnecessary' ERCP) and patient outcomes (cohort undergoing ERCP only).

Subgroup analyses will evaluate the primary outcome in patients defined by characteristics. The main subgroup analysis will be in patients with low versus moderate risk of CBD stones, which will be defined following phase 1. Further subgroups will be defined:

i. Patients referred for elective surgery versus patients undergoing urgent surgery;
ii. Patients with normal LFTs at baseline (i.e. low risk) versus patients with abnormal (outside of local Trust upper and/or lower normal limits) LFTs at baseline (i.e. moderate risk);
iii. Patients with a history of pancreatitis versus patients with no history of pancreatitis.

Health economic analysis

The primary economic evaluation will compare NHS costs and patient outcomes, measured by Quality Adjusted Life Years (QALYs), between the groups on an ITT basis. This analysis will explore whether the initial savings due to not using MRCP are offset by higher treatment costs and worse patient outcomes due to LC complications and/or retained stones.

English NHS tariffs will be used to estimate the costs.[17] These tariffs are based on Healthcare Resource Groups (HRGs) and distinguish between open/laparoscopic, elective/urgent and day case/inpatient surgery. However, they are not sufficiently granular to measure the impact on costs of small differences in theatre time or post-surgical length of stay which might be evident between groups. Therefore, micro-costing may also be used to estimate the incremental costs of LC. HRG codes, NHS tariffs and national unit costs will be used to estimate all other care costs during follow up.[18] National Institute for Health and Care Excellence recommended EQ-5D-5L value sets for

For peer review only

For peer review only - http://bmjopen.bmj.com/site/about/guidelines.xhtml

England will be used with linear interpolation between time-points and adjustment for baseline to calculate QALYs. [19, 20]

Costs and outcomes beyond 12 months will be discounted at standard rates.[21] Missing cost and EQ-5D-5L data will be described and multiple imputation techniques used as appropriate. The incremental cost per QALY of MRCP versus EM over the 18-month follow-up period will be estimated, and bootstrapping techniques used to estimate 95% confidence intervals. Regression analyses will be used to estimate the incremental net monetary benefit and cost-effectiveness acceptability curve of MRCP at conventional thresholds after controlling for key baseline covariates.[22] In a secondary economic analysis, we will estimate the cost per gallstone-related re-admission avoided. Sensitivity analyses will explore the robustness of the conclusions to plausible differences in key costing assumptions. If there is evidence that costs and outcome differences between groups persist between 6 and 18 months, a simple extrapolation model to estimate cost-effectiveness beyond 18 months will be considered. The impact of care pathways on patient productivity costs will be described in secondary analyses. A health economics analysis plan will detail analytical methods.

Safety

In accordance with Good Clinical Practice guidelines, the occurrence of adverse events will be monitored carefully and recorded in detail.

Following LC, transient post-operative complications are not unexpected and often delay the patient's hospital discharge. These complications are classified as expected events in this study. There are also some known complications of ERCP and MRCP which are also classified as expected events. Any event classified as expected will not require expedited reporting to the Sponsor or Research Ethics Committee (REC), unless in the event of a participant death. Unexpected serious

For peer review only - http://bmjopen.bmj.com/site/about/guidelines.xhtml

adverse events, including participant deaths, will be reported to the study Sponsor and coordinating centre.

Data on adverse events will be collected from randomisation until hospital discharge after LC, or for 9-months post-randomisation for participants who do not undergo LC for any reason.

Risk of Bias

Participating centres will aim to screen all adult patients with symptomatic gallbladder disease who are scheduled and fit for LC. We expect that the recruited patients will be generalisable to the wider population. Patients allocated to each group will have comparable baseline characteristics as randomisation will take place after consent via a secure website with allocation concealment. Allocations will be stratified by centre to minimise confounding.

The primary outcome is objective and will be obtained from routinely collected data sources. This is important as participants, clinicians and other hospital staff caring for participants will be unblinded. Attempts to blind participants by undertaking “dummy” MRCP in those randomised to EM would have added significant additional research costs and created logistical issues. Because “dummy” MRCP would cause a similar delay to LC as actual MRCP, it would also prevent the study from assessing the impact of this on outcomes.

The risk of missing data has been minimised by using routinely collected data for the primary outcome, and by undertaking regular review of missing data captured explicitly for the study.

We do not expect patient-reported outcomes to be at risk of bias, since we do not anticipate that participants will have preferences or expectations about the use of MRCP. We aim to promote uncertainty about the effects of the two interventions by providing information that describes the risks and benefits of each intervention. This information has been reviewed by a Public and Patient Involvement group and informed by the experiences of the QRI researchers.

For peer review only - http://bmjopen.bmj.com/site/about/guidelines.xhtml

For peer review only

Study management and oversight

Preparation of study documents, site initiation visits and training, day-to-day running of the study and monitoring of centres is managed by the Clinical Trials and Evaluation Unit, Bristol Trials Centre. A Study Executive Group (SEG) is overseeing the study and meets regularly to review milestones. A Data Monitoring and Safety Committee (DMSC) meets biannually to review data. A Study Steering Committee (SSC) oversees the study and has ultimate responsibility for any decision about its continuation. Membership of the study oversight committees is described in the Acknowledgments section.

Changes to the study protocol

Two substantial amendments have been approved by the REC. The first, in April 2019, added the option of postal consent and the second, in December 2019, revised some inclusion/exclusion criteria and clarified information relating to withdrawals in line with the Health Research Authority guidance.

DISCUSSION

The main challenge facing the Sunflower study is the recruitment target of 13,680 participants. Recruitment started 1 month later than planned in January 2019. The initial focus was to open centres to recruitment, with a target of 36 centres recruiting within phase 1. The study has already exceeded this target. As of 23rd August 2020, 48 centres were open to recruitment and 2,040 participants had been randomised. The QRI will be key to optimising recruitment, providing training at investigators meetings and recruitment tips for staff at participating centres.[23, 24] With a 48-month recruitment period, it will also be key for the study to keep centres engaged. Initiatives include the use of an active Twitter account, regular recruitment updates and centre targeted

For peer review only - http://bmjopen.bmj.com/site/about/guidelines.xhtml

emails, bi-annual newsletters and annual investigators' meetings. It will also be important to remain aware of other relevant research which could impact on the study. The study team will undertake annual literature reviews.

The study includes patients who are scheduled for LC as either an elective or urgent procedure. It is important to ensure that both pathways are represented to promote generalisability of the study results, but it is recognised that recruitment of urgent patients is challenging. These patients can be admitted at any time of day and over weekends when research nurses may not be available to approach patients and receive consent. As of 23rd August 2020, 77.8% of randomised participants were elective referrals and 22.2% were from urgent admissions. The study is keen to involve surgical trainees in the recruitment process, and Sunflower is participating in the National Institute for Health Research (NIHR) associate PI scheme.[25] During study training, the local team is encouraged to consider recruitment from the urgent pathway, and the ratio of elective to urgent patients recruited is being monitored. The study feels that surgical trainees will be critical to the successful recruitment of urgent patients. Discussions from an investigator meeting identified that the inclusion criterion, ALT and/or ALP less than 2xULN, was disproportionally excluding urgent patients and so this was amended to 3xULN. The impact of this amendment will be monitored.

For the study results to be generalisable, we aim to recruit both patients classified as being at low and moderate risk of CBD stones. Low risk patients are defined as those with bilirubin, ALT and ALP all within the normal range of their local Trust. As of 23rd August 2020, 67% of randomised participants were classified as being at low risk and 33% as moderate risk.

Another major challenge will be obtaining routinely collected data from England, Wales, Northern Ireland and Scotland. Applications to obtain routinely collected data are complex and time-consuming, and a different application process is required for each nation. The coordinating centre has considerable experience in completing these applications, but they must be successful and timely for the primary outcome to be determined and health economic analyses completed.

For peer review only

STUDY STATUS

Recruitment to the study started in January 2019 and is due to complete in January 2023. The current protocol is version 3.0 dated 25th November 2019. Relevant regulatory approvals will be obtained for amendments to the study documentation. Information relating to amendments will be disseminated to relevant parties via email, newsletter and an electronic study management system.

LIST OF ABBREVIATIONS

ALP: Alkaline phosphatase

ALT: Alanine transferase

CBD: Common bile duct

DMSC: Data Monitoring and Safety Committee

EM: Expectant management

ERCP: Endoscopic retrograde cholangiopancreatography

HES: Hospital Episode Statistics

HRG: Healthcare Resource Group

HRQoL: Health Related Quality of Life

ITT: Intention-to-treat

LC: Laparoscopic cholecystectomy

LFT: Liver function test

MRCP: Magnetic resonance cholangiopancreatography

NHS: National Health Service

For peer review only

For peer review only - http://bmjopen.bmj.com/site/about/guidelines.xhtml

NIHR: National Institute for Health Research

QALY: Quality Adjusted Life Year

QRI: QuinteT Recruitment Intervention

REC: Research Ethics Committee

SEG: Study Executive Group

SSC: Study Steering Committee

UK: United Kingdom

ULN: Upper limit of normal

USS: Ultrasound scan

ACKNOWLEDGEMENTS

The authors would like to thank all study staff involved in recruitment, coordination and data collection for this study, and members of the Patient and Public Involvement group who assisted with study design and review of study documents. Special thanks are also given to the clinical and nursing teams at participating centres for their support in the conduct of the study. The authors would like to acknowledge the DMSC and SSC for their oversight of the study. The independent DMSC is formed of a Chairperson, one consultant surgeon and one consultant radiologist. The SSC is formed of a Chairperson, two consultant surgeons, two consultant radiologists, one medical statistician and two public and patient involvement representatives; other SSC members with observer status represent the SEG in these committees.

The Sunflower SEG is made up of the following : Madeleine Clout, Jane Blazeby, Chris Rogers, Barnaby Reeves, Michelle Lazaroo, Kerry Avery, Natalie Blencowe, Ravinder Vohra, Neil Jennings,

For peer review only

William Hollingworth, Joanna Thorn, Marcus Jepson, Jane Collingwood, Ashley Guthrie, Elizabeth Booth, Samir Pathak, Ian Beckingham, Lucy Culliford, Ewen Griffiths, Raneem Albazaz and Giles Toogood.

The study is formally supported by the Association of Upper Gastrointestinal Surgeons of Great Britain and Ireland and the Great Britain & Ireland Hepato Pancreato Biliary Association, both of which recognise the importance of the objectives of this study.

DECLARATIONS

Ethics and dissemination

This study was reviewed and given a favourable opinion by Yorkshire & The Humber – South Yorkshire REC on the 10th December 2018 (REC reference: 18/YH/0358). Informed consent to participate will be obtained from all study participants. Participants have the right to withdraw from the study at any time and, if they do withdraw, will be treated according to standard local hospital procedures. Routine data will continue to be collected for withdrawn participants, unless the participant explicitly asks for this data collection to stop. Study registration number: ISRCTN10378861.

The study findings will be disseminated through peer-reviewed publications and international conferences, and provided to study participants. A study specific Twitter account will be used to promote the study, provide updates on milestones, and disseminate study findings. After study completion and reporting, the technical appendix, statistical code and dataset generated from the study will be available from the corresponding author on request. The request must include a specification of the data requested and justification for that request (i.e. a statement of purpose for which the data are required). The data may not be released unless all Bristol Trials Centre and Sponsor requirements are fulfilled (e.g. protocol, sample size calculation, REC and Health Research

For peer review only

Authority approval if appropriate, a collaboration arrangement in place between the Sponsor and the external body).

Competing interests

The authors declare that they have no competing interests.

Funding statement

This work is supported by the NIHR Health Technology Assessment Programme (Grant Ref: 16/142/04). The funder had no role in the design of the study, data collection or writing this manuscript. The study is also supported by the Royal College of Surgeons Surgical Trials Centre in Bristol. JMB, CR, BR, KA are all supported by the NIHR Bristol and Weston Biomedical Research Centre. JMB is a NIHR Senior Investigator. The study Sponsor is Leeds Teaching Hospitals NHS Trust (Research Governance Manager Anne Gowing, anne.gowing@nhs.net) and the study is managed by the Bristol Trials Centre, Clinical Trials and Evaluation Unit. This study was designed and is being delivered in collaboration with the Bristol Trials Centre, Clinical Trials and Evaluation Unit, a UK Clinical Research Collaboration registered clinical trials unit which is in receipt of NIHR clinical trials unit support funding. The research team acknowledges the support of the NIHR Clinical Research Network. The Sunflower study is overseen by an independent SSC and DMSC. The study has been designed with input from public and patient groups.

For peer review only

AUTHOR CONTRIBUTIONS

MC: Preparation and drafting of study protocol, first draft and writing of manuscript.

JMB: Study concept and design, preparation and review of study protocol, pre-study feasibility work, definition of study intervention and outcomes, review of manuscript.

For peer review only - http://bmjopen.bmj.com/site/about/guidelines.xhtml

CR: Study design, preparation and drafting of study protocol, sample size and statistical analysis plan, review of manuscript.

BR: Study concept and design, preparation and drafting of study protocol, review of manuscript.

ML: Statistical analyses, statistical analysis plan, review of manuscript.

KA: Study design, review of study protocol, leading PPI, review of manuscript.

NB: Study concept and design, review of study protocol, review of manuscript.

RV: Study concept and design, review of study protocol, review of manuscript.

NJ: Study concept and design, review of study protocol, review of manuscript.

WH: Study design, review of study protocol, health economic analysis plan and analyses, review of manuscript.

JT: Health economic analysis plan and analyses, review of manuscript.

MJ: Study design, review of study protocol, leading recruitment intervention, review of manuscript.

JC: Review of manuscript.

AG: Study design, review of study protocol, review of manuscript.

EB: Study design, review of study protocol, review of manuscript.

SP: Study design, review of study protocol, review of manuscript.

IB: Study concept and design, review of study protocol, review of manuscript.

LC: Study design, review of study protocol, review of manuscript.

EG: Study concept and design, review of study protocol, review of manuscript.

RA: Study concept and design, review of study protocol, review of manuscript.

For peer review only

For peer review only - http://bmjopen.bmj.com/site/about/guidelines.xhtml

568 GT: Study concept and design, preparation of study protocol, definition of study intervention and outcomes, review of manuscript. Chief investigator.

570 All authors read and approved the final manuscript.

571

572

For peer review only

For peer review only - http://bmjopen.bmj.com/site/about/guidelines.xhtml

REFERENCES

1. Sinha S, Hofman D, Stoker DL, et al. Epidemiological study of provision of cholecystectomy in England from 2000 to 2009: retrospective analysis of Hospital Episode Statistics. Surgical endoscopy. 2013;27(1):162-75.
2. Maple JT, Ikenberry SO, Anderson MA, et al. The role of endoscopy in the management of choledocholithiasis. Gastrointestinal endoscopy. 2011;74(4):731-44.
3. Costi R, Gnocchi A, Di Mario F, et al. Diagnosis and management of choledocholithiasis in the golden age of imaging, endoscopy and laparoscopy. World journal of gastroenterology. 2014;20(37):13382-401.
4. Treckmann J, Sauerland S, Frilling A, et al. Common Bile Duct Stones – Update 2006. In: Neugebauer EAM, Sauerland S, Fingerhut A, Millat B, Buess G, editors. EAES Guidelines for Endoscopic Surgery: Twelve Years Evidence-Based Surgery in Europe. Berlin, Heidelberg: Springer Berlin Heidelberg; 2006. p. 329-33.
5. Warttig S, Ward S, Rogers G. Diagnosis and management of gallstone disease: summary of NICE guidance. BMJ (Clinical research ed). 2014;349:g6241.
6. Williams EJ, Green J, Beckingham I, et al. Guidelines on the management of common bile duct stones (CBDS). Gut. 2008;57(7):1004-21.
7. CholeS Study Group WMRC. Population-based cohort study of outcomes following cholecystectomy for benign gallbladder diseases. Br J Surg. 2016;103(12):1704-15.
8. Noel R, Arnelo U, Lundell L, et al. Index versus delayed cholecystectomy in mild gallstone pancreatitis: results of a randomized controlled trial. HPB. 2018;20(10):932-8.
9. Anderson MA, Fisher L, Jain R, et al. Complications of ERCP. Gastrointestinal endoscopy. 2012;75(3):467-73.
10. Donovan JL, Rooshenas L, Jepson M, et al. Optimising recruitment and informed consent in randomised controlled trials: the development and implementation of the Quintet Recruitment Intervention (QRI). Trials. 2016;17(1):283.
11. Herbert A, Wijlaars L, Zylbersztejn A, et al. Data Resource Profile: Hospital Episode Statistics Admitted Patient Care (HES APC). International Journal of Epidemiology. 2017;46(4):1093-i.
12. EuroQoL Research Foundation. EQ-5D-5L User Guide: Basic information on how to use the EQ-5D-5L instrument2019 25 October 2019; 3.0. Available from: https://euroqol.org/wp-content/uploads/2019/09/EQ-5D-5L-English-User-Guide_version-3.0-Sept-2019-secured.pdf.
13. Gillies K. Development of core outcome set for symptomatic uncomplicated gallstone disease University of Aberdeen [Available from: https://www.abdn.ac.uk/hsru/what-we-do/research/projects/core-outcome-for-uncomplicated-gallstone-disease-398.php.
14. COMET Initiative. Development of core outcome set for symptomatic uncomplicated gallstone disease [Available from: http://www.comet-initiative.org/studies/details/927?result=true.
15. CONSORT Group. CONSORT Transparent Reporting of Trials [Available from: http://www.consort-statement.org/.
16. D'Agostino RB, Sr., Massaro JM, Sullivan LM. Non-inferiority trials: design concepts and issues - the encounters of academic consultants in statistics. Stat Med. 2003;22(2):169-86.
17. NHS England. National Tariff [Available from: https://www.england.nhs.uk/resources/pay-syst/national-tariff/.
18. Curtis LB, A. Unit Costs of Health and Social Care 2018. University of Kent; 2018 13 December 2018.
19. NICE. Position statement on use of the EQ-5D-5L value set for England (updated October 2019) 2019 [updated October 2019. Available from: https://www.nice.org.uk/about/what-we-do/our-programmes/nice-guidance/technology-appraisal-guidance/eq-5d-5l.
20. Manca A, Hawkins N, Sculpher MJ. Estimating mean QALYs in trial-based cost-effectiveness analysis: the importance of controlling for baseline utility. Health Econ. 2005;14(5):487-96.
21. Excellence NIfHaC. Guide to the methods of technology appraisal 2013 2013 [Available from: https://www.nice.org.uk/process/pmg9/chapter/foreword.

For peer review only - http://bmjopen.bmj.com/site/about/guidelines.xhtml

22. Hoch JS, Briggs AH, Willan AR. Something old, something new, something borrowed, something blue: a framework for the marriage of health econometrics and cost-effectiveness analysis. Health Econ. 2002;11(5):415-30.

23. Mills N, Blazeby JM, Hamdy FC, et al. Training recruiters to randomized trials to facilitate recruitment and informed consent by exploring patients' treatment preferences. Trials. 2014;15(1):323.

24. Paramasivan S, Rogers CA, Welbourn R, et al. Enabling recruitment success in bariatric surgical trials: pilot phase of the By-Band-Sleeve study. International Journal of Obesity. 2017;41(11):1654-61.

25. NIHR - National Institute for Health Research. Associate Principal Investigator (PI) Scheme 2019 [updated 30/05/2019. Available from: https://www.nihr.ac.uk/documents/associate-principal-investigator-pi-scheme/11694?diaryentryid=53625.

LEGENDS

Figure 1

LC = Laparoscopic cholecystectomy; MRCP = Magnetic resonance cholangiopancreatography

Table 1

[a] These timepoints are months post randomisation.

[b] During phase 1 of the study, some recruitment consultations will be audio-recorded at high volume centres with patient consent. Thereafter, some consultations will be recorded at centres where recruitment rates fall below target.

[c] Participants will not be asked to complete the laparoscopic cholecystectomy admission questionnaires if they have completed the baseline questionnaires within the previous two days.

For peer review only - http://bmjopen.bmj.com/site/about/guidelines.xhtml

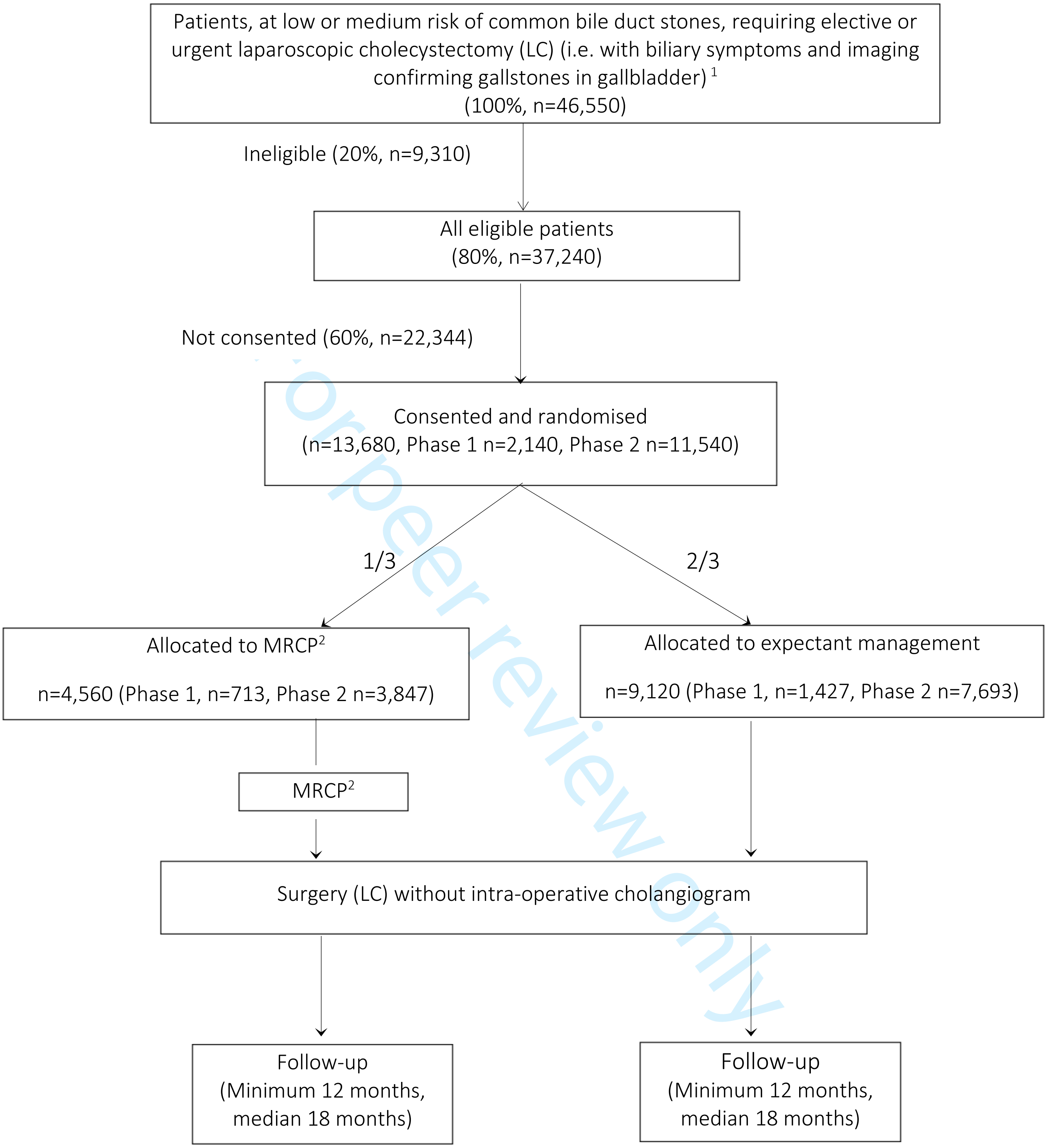

For peer review only

Reporting checklist for protocol of a clinical trial.

Based on the SPIRIT guidelines.

Instructions to authors

Complete this checklist by entering the page numbers from your manuscript where readers will find each of the items listed below.

Your article may not currently address all the items on the checklist. Please modify your text to include the missing information. If you are certain that an item does not apply, please write "n/a" and provide a short explanation.

Upload your completed checklist as an extra file when you submit to a journal.

In your methods section, say that you used the SPIRITreporting guidelines, and cite them as:

Chan A-W, Tetzlaff JM, Altman DG, Laupacis A, Gøtzsche PC, Krleža-Jerić K, Hróbjartsson A, Mann H, Dickersin K, Berlin J, Doré C, Parulekar W, Summerskill W, Groves T, Schulz K, Sox H, Rockhold FW, Rennie D, Moher D. SPIRIT 2013 Statement: Defining standard protocol items for clinical trials. Ann Intern Med. 2013;158(3):200-207

For peer review only

		Reporting Item	Page Number
**Administrative information**			
Title	#1	Descriptive title identifying the study design, population, interventions, and, if applicable, trial acronym	1
Trial registration	#2a	Trial identifier and registry name. If not yet registered, name of intended registry	6, 25
Trial registration: data set	#2b	All items from the World Health Organization Trial Registration Data Set	n/a, not a registry trial
Protocol version	#3	Date and version identifier	23
Funding	#4	Sources and types of financial, material, and other support	26
Roles and	#5a	Names, affiliations, and roles of protocol contributors	1-3

responsibilities: contributorship			
Roles and responsibilities: sponsor contact information	#5b	Name and contact information for the trial sponsor	26
Roles and responsibilities: sponsor and funder	#5c	Role of study sponsor and funders, if any, in study design; collection, management, analysis, and interpretation of data; writing of the report; and the decision to submit the report for publication, including whether they will have ultimate authority over any of these activities	26
Roles and responsibilities: committees	#5d	Composition, roles, and responsibilities of the coordinating centre, steering committee, endpoint adjudication committee, data management team, and other individuals or groups overseeing the trial, if applicable (see Item 21a for data monitoring committee)	24, 25
**Introduction**			
Background and rationale	#6a	Description of research question and justification for undertaking the trial, including summary of relevant studies (published and unpublished) examining benefits and harms for each intervention	8, 9
Background and rationale: choice of comparators	#6b	Explanation for choice of comparators	8, 9
Objectives	#7	Specific objectives or hypotheses	9
Trial design	#8	Description of trial design including type of trial (eg, parallel group, crossover, factorial, single group), allocation ratio, and framework (eg, superiority, equivalence, non-inferiority, exploratory)	10
**Methods: Participants, interventions, and outcomes**			

For peer review only

Study setting	#9	Description of study settings (eg, community clinic, academic hospital) and list of countries where data will be collected. Reference to where list of study sites can be obtained	10
Eligibility criteria	#10	Inclusion and exclusion criteria for participants. If applicable, eligibility criteria for study centres and individuals who will perform the interventions (eg, surgeons, psychotherapists)	10, 11
Interventions: description	#11a	Interventions for each group with sufficient detail to allow replication, including how and when they will be administered	15
Interventions: modifications	#11b	Criteria for discontinuing or modifying allocated interventions for a given trial participant (eg, drug dose change in response to harms, participant request, or improving / worsening disease)	25
Interventions: adherance	#11c	Strategies to improve adherence to intervention protocols, and any procedures for monitoring adherence (eg, drug tablet return; laboratory tests)	10
Interventions: concomitant care	#11d	Relevant concomitant care and interventions that are permitted or prohibited during the trial	10, 11
Outcomes	#12	Primary, secondary, and other outcomes, including the specific measurement variable (eg, systolic blood pressure), analysis metric (eg, change from baseline, final value, time to event), method of aggregation (eg, median, proportion), and time point for each outcome. Explanation of the clinical relevance of chosen efficacy and harm outcomes is strongly recommended	11-13
Participant timeline	#13	Time schedule of enrolment, interventions (including any run-ins and washouts), assessments, and visits for participants. A schematic diagram is highly recommended (see Figure)	13
Sample size	#14	Estimated number of participants needed to achieve study objectives and how it was determined, including clinical and statistical assumptions supporting any	14

For peer review only

		sample size calculations	
Recruitment	#15	Strategies for achieving adequate participant enrolment to reach target sample size	14
**Methods: Assignment of interventions (for controlled trials)**			
Allocation: sequence generation	#16a	Method of generating the allocation sequence (eg, computer-generated random numbers), and list of any factors for stratification. To reduce predictability of a random sequence, details of any planned restriction (eg, blocking) should be provided in a separate document that is unavailable to those who enrol participants or assign interventions	15
Allocation concealment mechanism	#16b	Mechanism of implementing the allocation sequence (eg, central telephone; sequentially numbered, opaque, sealed envelopes), describing any steps to conceal the sequence until interventions are assigned	15
Allocation: implementation	#16c	Who will generate the allocation sequence, who will enrol participants, and who will assign participants to interventions	15
Blinding (masking)	#17a	Who will be blinded after assignment to interventions (eg, trial participants, care providers, outcome assessors, data analysts), and how	20
Blinding (masking): emergency unblinding	#17b	If blinded, circumstances under which unblinding is permissible, and procedure for revealing a participant's allocated intervention during the trial	n/a, no blinding
**Methods: Data collection, management, and analysis**			
Data collection plan	#18a	Plans for assessment and collection of outcome, baseline, and other trial data, including any related processes to promote data quality (eg, duplicate	16

For peer review only

		measurements, training of assessors) and a description of study instruments (eg, questionnaires, laboratory tests) along with their reliability and validity, if known. Reference to where data collection forms can be found, if not in the protocol	
Data collection plan: retention	#18b	Plans to promote participant retention and complete follow-up, including list of any outcome data to be collected for participants who discontinue or deviate from intervention protocols	25
Data management	#19	Plans for data entry, coding, security, and storage, including any related processes to promote data quality (eg, double data entry; range checks for data values). Reference to where details of data management procedures can be found, if not in the protocol	16
Statistics: outcomes	#20a	Statistical methods for analysing primary and secondary outcomes. Reference to where other details of the statistical analysis plan can be found, if not in the protocol	17
Statistics: additional analyses	#20b	Methods for any additional analyses (eg, subgroup and adjusted analyses)	17-19
Statistics: analysis population and missing data	#20c	Definition of analysis population relating to protocol non-adherence (eg, as randomised analysis), and any statistical methods to handle missing data (eg, multiple imputation)	17-19
**Methods: Monitoring**			
Data monitoring: formal committee	#21a	Composition of data monitoring committee (DMC); summary of its role and reporting structure; statement of whether it is independent from the sponsor and competing interests; and reference to where further details about its charter can be found, if not in the protocol. Alternatively, an explanation of why a DMC is not needed	24
Data monitoring:	#21b	Description of any interim analyses and stopping	17

For peer review only

interim analysis		guidelines, including who will have access to these interim results and make the final decision to terminate the trial	
Harms	#22	Plans for collecting, assessing, reporting, and managing solicited and spontaneously reported adverse events and other unintended effects of trial interventions or trial conduct	19, 20
Auditing	#23	Frequency and procedures for auditing trial conduct, if any, and whether the process will be independent from investigators and the sponsor	15
**Ethics and dissemination**			
Research ethics approval	#24	Plans for seeking research ethics committee / institutional review board (REC / IRB) approval	25
Protocol amendments	#25	Plans for communicating important protocol modifications (eg, changes to eligibility criteria, outcomes, analyses) to relevant parties (eg, investigators, REC / IRBs, trial participants, trial registries, journals, regulators)	23
Consent or assent	#26a	Who will obtain informed consent or assent from potential trial participants or authorised surrogates, and how (see Item 32)	14
Consent or assent: ancillary studies	#26b	Additional consent provisions for collection and use of participant data and biological specimens in ancillary studies, if applicable	n/a
Confidentiality	#27	How personal information about potential and enrolled participants will be collected, shared, and maintained in order to protect confidentiality before, during, and after the trial	16
Declaration of interests	#28	Financial and other competing interests for principal investigators for the overall trial and each study site	26
Data access	#29	Statement of who will have access to the final trial dataset, and disclosure of contractual agreements that	25, 26

For peer review only

		limit such access for investigators	
Ancillary and post trial care	#30	Provisions, if any, for ancillary and post-trial care, and for compensation to those who suffer harm from trial participation	Not included
Dissemination policy: trial results	#31a	Plans for investigators and sponsor to communicate trial results to participants, healthcare professionals, the public, and other relevant groups (eg, via publication, reporting in results databases, or other data sharing arrangements), including any publication restrictions	25
Dissemination policy: authorship	#31b	Authorship eligibility guidelines and any intended use of professional writers	26-28
Dissemination policy: reproducible research	#31c	Plans, if any, for granting public access to the full protocol, participant-level dataset, and statistical code	25
**Appendices**			
Informed consent materials	#32	Model consent form and other related documentation given to participants and authorised surrogates	Available on request
Biological specimens	#33	Plans for collection, laboratory evaluation, and storage of biological specimens for genetic or molecular analysis in the current trial and for future use in ancillary studies, if applicable	n/a, no specimens

None The SPIRIT checklist is distributed under the terms of the Creative Commons Attribution License CC-BY-ND 3.0. This checklist can be completed online using https://www.goodreports.org/, a tool made by the EQUATOR Network in collaboration with Penelope.ai

For peer review only

BMJ Open

Study protocol for a randomised controlled trial to establish the clinical and cost effectiveness of expectant management versus pre-operative imaging with magnetic resonance cholangiopancreatography in patients with symptomatic gallbladder disease undergoing laparoscopic cholecystectomy at low or moderate risk of common bile duct stones (The Sunflower Study)

Journal:	*BMJ Open*
Manuscript ID	bmjopen-2020-044281.R1
Article Type:	Protocol
Date Submitted by the Author:	01-Feb-2021

Complete List of Authors:	Clout, Madeleine; University of Bristol Faculty of Medical and Veterinary Sciences, Clinical Trials and Evaluation Unit<br>Blazeby, Jane; University of Bristol, Department of Social Medicine<br>Rogers, Chris; university of bristol, Clinical Science South Bristol<br>Reeves, Barnaby; University of Bristol, Bristol Heart Institute<br>Lazaroo, Michelle; University of Bristol Faculty of Medical and Veterinary Sciences, Clinical Trials and Evaluation Unit<br>Avery, Kerry; University of Bristol, School of Social and Community Medicine<br>Blencowe, Natalie; University of Bristol, Centre for Surgical Research, School of Social and Community Medicine<br>Vohra, Ravi; Nottingham City Hospital<br>Jennings, Neil; Sunderland Royal Hospital<br>Hollingworth, William; University of Bristol, School of Social and Community Medicine<br>Thorn, Joanna; University of Bristol, School of Social and Community Medicine<br>Jepson, Marcus; University of Bristol School of Social and Community Medicine,<br>Collingwood, Jane; University of Bristol, School of Population Health Sciences<br>Guthrie, Ashley; Leeds Teaching Hospitals NHS Trust<br>Booth, Elizabeth; NHS Coventry and Rugby Clinical Commissioning Group<br>Pathak, Samir; University of Bristol, Centre for Surgical Research, Population Health Sciences<br>Beckingham, Ian; Nottingham University Hospitals NHS Trust<br>Culliford, Lucy; University of Bristol, Bristol Heart Institute,<br>Griffiths, Ewen; University Hospitals Birmingham NHS Foundation Trust, Department of Upper GI Surgery<br>Albazaz, Raneem; Leeds Teaching Hospitals NHS Trust<br>Toogood, Giles; Leeds Teaching Hospitals NHS Trust

**Primary Subject Heading**:	Surgery

Secondary Subject Heading:	Gastroenterology and hepatology, Radiology and imaging, Health economics, Qualitative research
Keywords:	Magnetic resonance imaging < RADIOLOGY & IMAGING, Endoscopy < GASTROENTEROLOGY, SURGERY, GASTROENTEROLOGY, NEPHROLOGY, Clinical trials < THERAPEUTICS

SCHOLARONE™
Manuscripts

I, the Submitting Author has the right to grant and does grant on behalf of all authors of the Work (as defined in the below author licence), an exclusive licence and/or a non-exclusive licence for contributions from authors who are: i) UK Crown employees; ii) where BMJ has agreed a CC-BY licence shall apply, and/or iii) in accordance with the terms applicable for US Federal Government officers or employees acting as part of their official duties; on a worldwide, perpetual, irrevocable, royalty-free basis to BMJ Publishing Group Ltd ("BMJ") its licensees and where the relevant Journal is co-owned by BMJ to the co-owners of the Journal, to publish the Work in this journal and any other BMJ products and to exploit all rights, as set out in our licence.

The Submitting Author accepts and understands that any supply made under these terms is made by BMJ to the Submitting Author unless you are acting as an employee on behalf of your employer or a postgraduate student of an affiliated institution which is paying any applicable article publishing charge ("APC") for Open Access articles. Where the Submitting Author wishes to make the Work available on an Open Access basis (and intends to pay the relevant APC), the terms of reuse of such Open Access shall be governed by a Creative Commons licence – details of these licences and which Creative Commons licence will apply to this Work are set out in our licence referred to above.

Other than as permitted in any relevant BMJ Author's Self Archiving Policies, I confirm this Work has not been accepted for publication elsewhere, is not being considered for publication elsewhere and does not duplicate material already published. I confirm all authors consent to publication of this Work and authorise the granting of this licence.

For peer review only

Study protocol for a randomised controlled trial to establish the clinical and cost effectiveness of expectant management versus pre-operative imaging with magnetic resonance cholangiopancreatography in patients with symptomatic gallbladder disease undergoing laparoscopic cholecystectomy at low or moderate risk of common bile duct stones (The Sunflower Study)

On behalf of the Sunflower Study Executive Group*

*Authors for 'Sunflower Study Executive Group':

1. Madeleine Clout, Clinical Trials and Evaluation Unit, Bristol Trials Centre, University of Bristol, Bristol Royal Infirmary, Zone A Level 7, Bristol, BS2 8HW, UK, madeleine.clout@bristol.ac.uk
2. Jane Blazeby, NIHR Bristol Biomedical Research Centre, Centre for Surgical Research, Bristol Medical School, Population Health Sciences, University of Bristol, Office 4.05 Canynge Hall, 39 Whatley Road, Bristol, BS8 2PS, UK, j.m.blazeby@bristol.ac.uk
3. Chris Rogers, Clinical Trials and Evaluation Unit, Bristol Trials Centre, University of Bristol, Bristol Royal Infirmary, Zone A Level 7, Bristol, BS2 8HW, UK, chris.rogers@bristol.ac.uk
4. Barnaby C Reeves, Clinical Trials and Evaluation Unit, Bristol Trials Centre, University of Bristol, Bristol Royal Infirmary, Zone A Level 7, Bristol, BS2 8HW, UK, barney.reeves@bristol.ac.uk
5. Michelle Lazaroo, Clinical Trials and Evaluation Unit, Bristol Trials Centre, University of Bristol, Bristol Royal Infirmary, Zone A Level 7, Bristol, BS2 8HW, UK, michelle.lazaroo@bristol.ac.uk
6. Kerry Avery, NIHR Bristol Biomedical Research Centre and Centre for Surgical Research Bristol Medical School, Population Health Sciences, University of Bristol, 39 Whatley Road, Bristol, BS8 2PS, UK, Kerry.Avery@bristol.ac.uk

For peer review only

For peer review only - http://bmjopen.bmj.com/site/about/guidelines.xhtml

7. Natalie Blencowe, NIHR Bristol Biomedical Research Centre and Centre for Surgical Research, Population Health Sciences, Bristol Medical School, University of Bristol, 39 Whatley Road, Bristol, BS8 2PS, UK, Natalie.Blencowe@bristol.ac.uk
8. Ravi Vohra, Trent Oesophago-Gastric Unit, Nottingham University Hospitals NHS Trust, City Hospital, Hucknall Rd, Nottingham, NG5 1PB, UK, ravinder.vohra@nuh.nhs.com
9. Neil Jennings, Sunderland Royal Hospital, Kayll Road, Sunderland, Tyne & Wear, SR4 7TP, UK, Neil.Jennings@chsft.nhs.uk
10. William Hollingworth, Bristol Medical School, Population Health Sciences, University of Bristol, Office 2.08, 1-5 Whiteladies Road, Bristol, BS8 1NU, UK, william.hollingworth@bristol.ac.uk
11. Joanna Thorn, Bristol Medical School, Population Health Sciences, University of Bristol, Office 2.12, 1-5 Whiteladies Road, Bristol, BS8 1NU, UK, joanna.thorn@bristol.ac.uk
12. Marcus Jepson, Bristol Medical School, Population Health Sciences, University of Bristol, Office 4.03 Canynge Hall, 39 Whatley Road, Bristol, BS8 2PS, UK, marcus.jepson@bristol.ac.uk
13. Jane Collingwood, Bristol Medical School, Population Health Sciences, University of Bristol, Office 2.01 Canynge Hall, 39 Whatley Road, Bristol, BS8 2PS, UK, jane.collingwood@bristol.ac.uk
14. Ashley Guthrie, Leeds Teaching Hospitals NHS Trust, St James's University Hospital, Beckett Street, Leeds, LS9 7TF, UK, ashley.guthrie@nhs.net
15. Elizabeth Booth, NHS Coventry and Rugby Clinical Commissioning Group, Parkside House, Quinton Road, Coventry, CV1 2NJ, UK, elizabeth.booth@coventryrugbyccg.nhs.uk
16. Samir Pathak, Centre for Surgical Research and Centre for Surgical Research, Population Health Sciences, Bristol Medical School, University of Bristol, 39 Whatley Road, Bristol, BS8 2PS, UK, drsamirpathak@gmail.com

For peer review only

For peer review only - http://bmjopen.bmj.com/site/about/guidelines.xhtml

17. Ian Beckingham, Nottingham University Hospitals NHS Trust, Hucknall Road, Nottingham, NG5 1PB, UK, ian.beckingham@nuh.nhs.uk
18. Lucy Culliford, Clinical Trials & Evaluation Unit, Bristol Trials Centre, University of Bristol, Bristol Royal Infirmary, Zone A Level 7, Bristol, BS2 8HW, UK, lucy.culliford@bristol.ac.uk
19. Ewen A Griffiths, University Hospitals Birmingham NHS Foundation Trust, Queen Elizabeth Hospital Birmingham, Mindelsohn Way, Edgbaston, Birmingham, B15 2GW, UK, Ewen.Griffiths@uhb.nhs.uk
20. Raneem Albazaz, Leeds Teaching Hospitals NHS Trust, St James's University Hospital, Beckett Street, Leeds, LS9 7TF, UK, r.albazaz@nhs.net
21. Giles Toogood, Department of Hepatobiliary and Transplantation Surgery, Leeds Teaching Hospitals NHS Trust, St James's University Hospital, Beckett Street, Leeds, LS9 7TF, UK, giles.toogood@nhs.net

Corresponding author

Mrs Madeleine Clout

madeleine.clout@bristol.ac.uk

Clinical Trials and Evaluation Unit

Bristol Trials Centre

University of Bristol

Bristol Royal Infirmary, Zone A Level 7

Bristol

BS2 8HW

0117 342 2526 / 07929 771395

For peer review only

For peer review only - http://bmjopen.bmj.com/site/about/guidelines.xhtml

Word count: 4512

Keywords: Gallstones, gallbladder disease, laparoscopic cholecystectomy, magnetic resonance cholangiopancreatography, randomised controlled trial, common bile duct, endoscopic retrograde cholangiopancreatography

For peer review only

ABSTRACT:

Introduction

Surgery to remove the gallbladder (laparoscopic cholecystectomy (LC)) is the standard treatment for symptomatic gallbladder disease. One potential complication of gallbladder disease is that gallstones can pass into the common bile duct (CBD) where they may remain dormant, pass spontaneously into the bowel or cause problems such as obstructive jaundice or pancreatitis. Patients requiring LC are assessed pre-operatively for their risk of CBD stones using liver function tests and imaging. If the risk is high, guidelines recommend further investigation and treatment. Further investigation of patients at low or moderate risk of CBD stones is not standardised, and the practice of imaging the CBD using magnetic resonance cholangiopancreatography (MRCP) in these patients varies across the United Kingdom (UK). The consequences of these decisions may lead to over or under treatment of patients.

Methods and analysis

We are conducting a UK multicentre, pragmatic, open, randomised controlled trial with internal pilot phase to compare the effectiveness and cost-effectiveness of pre-operative imaging with MRCP versus expectant management (i.e. no pre-operative imaging) in adult patients with symptomatic gallbladder disease undergoing urgent or elective LC who are at low or moderate risk of CBD stones. We aim to recruit 13,680 patients over 48 months. The primary outcome is any hospital admission within 18 months of randomisation for a complication of gallstones. This includes complications of endoscopic retrograde cholangiopancreatography for the treatment of gallstones, and complications of LC. This will be determined using routine data sources, e.g. NHS Digital Hospital Episode Statistics for participants in England. Secondary outcomes include cost-effectiveness and patient-reported quality of life, with participants followed up for a median of 18 months.

Ethics and dissemination

For peer review only

For peer review only - http://bmjopen.bmj.com/site/about/guidelines.xhtml

104 This study received approval from Yorkshire & The Humber – South Yorkshire Research Ethics Committee. Results will be submitted for publication in a peer-reviewed journal.

106 **Registration details**: ISRCTN10378861

For peer review only

For peer review only - http://bmjopen.bmj.com/site/about/guidelines.xhtml

ARTICLE SUMMARY

Strengths and limitations of this study

- This is a randomised controlled trial with randomisation taking place using a secure internet-based system to ensure allocation concealment, which will ensure that patients allocated to each group have comparable baseline characteristics.
- The study includes an internal pilot phase with progression to the main study dependent on a number of criteria, to ensure that the study will only proceed if it is viable to do so.
- Participants, clinicians and other hospital staff caring for participants will not be blind to the study allocation.
- The criteria used to define low and moderate risk of common bile duct stones have been identified by clinicians within the study team, as there is no universal agreed definition of these terms.
- The study primary outcome will be derived from routine data sources, which should ensure high levels of data completeness but is reliant on timely approval of numerous applications to holders of routine data.

For peer review only

For peer review only - http://bmjopen.bmj.com/site/about/guidelines.xhtml

INTRODUCTION

Surgery to remove the gallbladder, known as laparoscopic cholecystectomy (LC), is the standard treatment for symptomatic gallbladder disease. LC is one of the most common operations undertaken globally and around 70,000 LC operations are performed annually in England.[1]

One potential complication of gallbladder disease is that gallstones may pass from the gallbladder into the common bile duct (CBD). Once in the CBD, they may remain dormant, pass spontaneously into the bowel or cause problems such as obstructive jaundice, cholangitis or pancreatitis. If CBD stones are found, current guidelines recommend removal either before or during LC. Therefore, patients requiring LC are assessed pre-operatively for their risk of CBD stones using a combination of liver function tests (LFTs) and imaging. If the risk is high, guidelines recommend further investigation and treatment.[2-6] Further investigation of patients at moderate or low risk of CBD stones is not standardised and practice across the United Kingdom (UK) varies. Fewer than 10% of these patients actually have CBD stones.[2-4, 6, 7] Consequently, surgeons make different decisions about investigation of the CBD; some surgeons choose to perform diagnostic imaging tests for these patients, and others do not. This variation likely results in over or under treatment, with significant risks to the patient in terms of morbidity, Health Related Quality of Life (HRQoL) and costs to the health service.

A UK-wide audit found that a third of patients undergoing LC, irrespective of risk of CBD stones, had diagnostic imaging to test for CBD stones.[7] This is most frequently performed with magnetic resonance cholangiopancreatography (MRCP) before admission for LC, however some surgeons instead use intra-operative cholangiogram and other intra-operative ultrasound examinations of the CBD. MRCP produces detailed images of the biliary and pancreatic ducts, involves a 1-hour hospital visit and costs the National Health Service (NHS) about £365. MRCP can identify CBD stones but may delay LC, which can have a detrimental effect on HRQoL and can lead to increased problems with gallstones or a more complex LC.[8] If a CBD stone is identified during MRCP, extraction is most

For peer review only - http://bmjopen.bmj.com/site/about/guidelines.xhtml

For peer review only

frequently performed before LC with an endoscopic retrograde cholangiopancreatography (ERCP). The risks and mortality associated with ERCP are significant,[9] as is the cost to the NHS (about £1,600). Not having MRCP avoids these risks but can lead to CBD stones remaining after surgery, which may also cause complications. Research is needed to establish whether it is appropriate for patients at low and moderate risk of CBD stones to proceed straight to LC without imaging the CBD with MRCP.

METHODS AND ANALYSIS

Aims and Objectives

The Sunflower study aims to compare the effectiveness and cost-effectiveness of pre-operative imaging with MRCP versus no MRCP (hereafter called expectant management (EM)) in patients with symptomatic gallbladder disease undergoing LC who are at low or moderate risk of CBD stones. The study will test the hypothesis that EM is not inferior to MRCP with respect to hospitalisation for treatment for a complication of gallstones up to 18 months after randomisation.

The objectives are to estimate the:

1. Difference between groups in the proportion of participants requiring a hospital admission for treatment of a complication of gallstones in the gallbladder or CBD.
2. Difference between groups with respect to a range of secondary outcomes, including patient-reported HRQoL, symptoms related to complications of gallstones in the gallbladder or CBD, and symptoms related to complications associated with LC and, where applicable, ERCP.
3. Cost-effectiveness of MRCP compared with EM.

For peer review only - http://bmjopen.bmj.com/site/about/guidelines.xhtml

Study Design

The Sunflower study is a multi-centre, pragmatic, open, randomised controlled trial with an internal pilot. The study has an embedded Qualitative Research Integrated within Trials Recruitment Intervention (QRI) to optimise informed consent and recruitment.[10]

There are two phases to the study:

Phase 1 (internal pilot): Set-up and recruit from at least 36 centres by month 16 of recruitment, with integrated QRI to optimise recruitment.

Phase 2: Increase the number of centres to at least 50 and continue recruitment using the methods established in Phase 1, along with integrated QRI to optimise recruitment and promote adherence in new centres.

Progression to phase 2 is conditional on the study demonstrating to the funder that: a) at least 30 centres are open and recruiting; b) at least 1,750 participants have been randomised; c) at least 90% of participants have followed their allocated pathway; and d) the primary outcome can be identified reliably from routine data.

Setting

Recruitment will take place at a minimum of 50 NHS hospitals across the UK with a recruitment target of 13,680 participants over a 48-month period (

Figure **1**).

Study Population

The target population is adults (18 years or older) with symptomatic gallbladder disease, scheduled and fit for LC as an elective or urgent procedure, who are at low or moderate risk of CBD stones. Low

For peer review only

or moderate risk is defined as CBD diameter ≤8mm on ultrasound scan (USS), and bilirubin ≤50umol/l, and alanine transferase (ALT) less than three times the upper limit of normal (≤3xULN) and/or alkaline phosphatase (ALP) ≤3xULN. If the CBD cannot be visualised on USS, the patient may be recruited if no intrahepatic duct dilatation is reported.

Patients are excluded if they are unable to undergo MRCP, have evidence of empyema or perforated gallbladder requiring urgent intervention, have had a previous gastric bypass, have had a previous MRCP or endoscopic USS within the last 3 months, have had any previous ERCP or have haemolytic disease. Patients must be willing to participate in follow up and be able to provide informed consent.

Figure 1: The study schema

Primary Outcome

The primary outcome is any of the following:

i. Any hospital admission within 18 months of randomisation for treatment of a complication of gallstones whether in the CBD or gallbladder;
ii. Complications during the admission for LC for the treatment for gallstones or any readmission for complications of the LC leading to a hospital day of >2 days. Complications will include, but not be limited to, return to theatre post LC for any cause, percutaneous radiological drainage and ERCP for non-diagnostic reasons (e.g. for a bile leak). It does not include a diagnostic ERCP performed following an MRCP where CBD stones were identified;
iii. Complications during any ERCP for the treatment for gallstones. Complications will include, but are not limited to, blood transfusion post ERCP, percutaneous radiological drainage, treatment of a perforation occurring during ERCP, acute pancreatitis and other complications leading to a hospital stay of > 2 days.

For peer review only - http://bmjopen.bmj.com/site/about/guidelines.xhtml

This outcome will be ascertained by linking the study data with routinely-collected Hospital Episode Statistics (HES) and mortality data from NHS Digital for participants recruited in England, and the equivalent data sources for participants recruited in Scotland, Wales and Northern Ireland. Routine data sources contain information on care provided to all patients treated in UK NHS hospitals.[11] The final specification of qualifying events for identifying the primary outcome from these routine sources will be developed and validated during phase 1 of the study, through comparison of routine data with events identified from review of the medical records at 90 days post-LC.

Secondary Outcomes

Patient-reported HRQoL will be measured using the EQ-5D-5L questionnaire.[12] To minimise the overall study burden, about 20% of participants will be sampled and asked to complete questionnaires at multiple timepoints (Table 1). A questionnaire pack will be provided to participants containing the validated EQ-5D-5L questionnaire plus questions about the impact of abdominal pain on work, productivity and primary healthcare use.

For peer review only

For peer review only - http://bmjopen.bmj.com/site/about/guidelines.xhtml

Data item	Pre-Randomisation	Pre-Surgery	Hospital admission for LC	90 days post LC	3 - months[a]	6-months[a]	12-months[a]	18-months[a]
Eligibility	✓							
Written informed consent	✓							
Medical history	✓							
EQ-5D-5L, productivity & primary care use questionnaire (20% sample)	✓		✓[c]		✓	✓	✓	✓
MRCP, ERCP and intraoperative imaging details, if applicable		✓						
Operative and post-operative details			✓					
Items in the symptomatic gallstone core outcome set				✓				
Safety data				✓				
Study consultations audio recorded for QRI [b]	✓							

Table 1: Data collection time points

Other secondary outcomes include items that cover the domains in the core outcome set for patients with symptomatic gallstones, and hospital resource use in the 18 months after randomisation collected from routine data sources.[13, 14]

For peer review only

For peer review only - http://bmjopen.bmj.com/site/about/guidelines.xhtml

Sample Size

The sample size of 13,680 has been chosen to test the hypothesis that EM is non-inferior to MRCP with respect to the primary outcome. In estimating the sample size, we have considered the proportion of patients that would be expected to experience the primary outcome, as identified in the CholeS audit (5% to 10%) and through exploration of HES data.[7] This includes patients at high risk of CBD stones. The consensus amongst clinicians on the study team was that the non-inferiority margin should be set at 1.5%, i.e. that the risk of the primary outcome with EM should not exceed 8.5% assuming a risk of 7% after MRCP. A study of 13,680 participants will provide 90% power to test the non-inferiority hypothesis for a 7% event risk with MRCP and 80% power to test the non-inferiority hypothesis for a higher event risk of 8.5%., assuming 2.5% one-sided statistical significance and a 1:2 allocation ratio (MRCP:EM). The 20% sample of participants providing HRQoL data will be stratified by centre. This sample of 2,736 participants will have >90% power to detect a small effect size of 0.12 standard deviations between groups on the EQ-5D-5L questionnaire.

Recruitment

Recruiting centres will screen LC waiting lists and gallbladder clinic lists for potentially eligible patients. Potential participants will also be identified from urgent inpatient and ambulatory care admissions and ward handovers. Eligible patients will be provided with study information and will have the opportunity to ask questions before being approached for their informed consent. Patients who feel they have had insufficient time to consider the study will be invited to take home a consent form and questionnaire pack (where applicable), and to complete and return them by post. Details of all patients approached for the study and reasons for non-participation will be documented.

For peer review only - http://bmjopen.bmj.com/site/about/guidelines.xhtml

Randomisation

Randomisation will be performed by an authorised member of the local research team using a secure internet-based system to ensure allocation concealment. The allocation will be computer generated and stratified by centre.

Participants will be randomised to MRCP or EM in a 1:2 ratio. This ratio has been selected to reflect current levels of MRCP provision (13-26% of patients at low or moderate risk of CBD stones currently undergo MRCP).[7]

Interventions

The study interventions are pre-operative MRCP and EM.

Participants allocated to the MRCP group will have an MRCP before the date of their listed LC. The study will make no changes to the usual hospital radiology protocols used for MRCP and will not impose any timelines for the MRCP, or any subsequent interventions. If a CBD stone is found on MRCP, surgeons can manage extraction according to their usual practice. The study does not stipulate removal of identified CBD stones, and any further interventions are at the discretion of the clinician. If an ERCP is requested, it will be performed as a separate inpatient procedure before admission for LC.

Participants allocated to EM will be listed for LC without any pre-operative imaging. If imaging is carried out, e.g. due to a change in a participant's clinical circumstances after randomisation, this will be documented as a crossover.

In both groups, intraoperative imaging will not be permitted unless there is an anatomical reason to do so, or if a CBD stone is found on MRCP. Any deviations from the protocol will be recorded and monitored.

For peer review only - http://bmjopen.bmj.com/site/about/guidelines.xhtml

Data collection

Data will be collected by the local study team at baseline, before, during and after LC, and at hospital discharge (Table 1). Data collection will be from hospital records, including operative notes, and will include information on any pre-LC MRCP and ERCP, and any intra-operative imaging. Data will either be collected onto a paper case report form and transcribed into a secure electronic study database or entered directly into the database. During phase 1, safety data will also be collected from hospital records 90 days post-LC by the local study team.

Linkage to routine data

With consent, participant identifiers will be sent to NHS Digital, and equivalent organisations for participants recruited outside of England. These organisations will match and extract pseudonymised data for these participants for all episodes of hospital care for a minimum of 18 months from the point of randomisation, and for a period of 12 months prior to randomisation. The data will include inpatient and critical care episodes, accident and emergency department attendances, out-patient appointments, diagnostic imaging investigations and mortality.

MRCP Quality Assurance

To address the challenge of MRCP technique varying across participating centres, images and reports for a random 10% sample of participants who receive an MRCP will be transferred for independent quality assurance review by consultant radiologists. The MRCP examinations will be assessed for the type of sequences performed, the diagnostic quality of the images and level of concordance with the hospital reports. This data will provide an overview of current NHS practice and the overall quality of MRCP examinations, as well as characterising variation in MRCP technique in study centres.

For peer review only - http://bmjopen.bmj.com/site/about/guidelines.xhtml

Statistical analysis

The study will be analysed on an intention-to-treat (ITT) basis, i.e. outcomes will be analysed according to the allocated treatment pathway irrespective of future management and events, and every effort will be made to include all randomised participants. Non-adherence to the treatment allocation will be documented. The primary analysis will follow the Consolidated Standards of Reporting Trials guidelines for a non-inferiority study.[15] An analysis according to treatment pathway followed will also be performed for the primary outcome. As recommended, both analyses will be considered when assessing whether the hypothesis is met.[16] The primary outcome will be compared using survival methods to allow for censoring. For participants without a qualifying primary outcome event, they will be censored at the time the dataset was compiled. If more than one qualifying event occurs (e.g. two hospital admissions for gallstone-related complications), time to the first event will be used. The frequency of each element of the primary outcome and reasons for admission, where applicable, will be described. Secondary outcomes will be compared using a mixed model for continuous outcomes or a generalised linear regression model for binary outcomes, adjusted for pre-randomisation measures when available. For secondary outcomes measured at multiple time points, changes in treatment effect with time since randomisation will be assessed by adding a treatment by time interaction to the model and comparing models using a likelihood ratio test. Model fit will be assessed and alternative models and/or transformations (e.g. to induce normality) will be explored where appropriate. Sensitivity analyses using multiple imputation for missing data will be explored. Analyses will be adjusted for centre and treatment differences will be reported with 95% confidence intervals.

A detailed analysis plan will be prepared. There is no intention to compare any outcomes between groups at the end of phase 1; only descriptive statistics about eligibility, recruitment and adherence will be summarised at this time to determine whether the study satisfies the progression criteria.

Exploratory analyses will include:

For peer review only - http://bmjopen.bmj.com/site/about/guidelines.xhtml

i. Relationship between number and size of stones seen on MRCP and patient outcome (cohort undergoing MRCP only);
ii. Relationship between stones removed under ERCP or not (e.g., 'necessary' vs. 'unnecessary' ERCP) and patient outcomes (cohort undergoing ERCP only).

Subgroup analyses will evaluate the primary outcome in patients defined by characteristics. The main subgroup analysis will be in patients with low versus moderate risk of CBD stones, which will be defined following phase 1. This definition is expected to be a composite of baseline LFTs, baseline CBD diameter on USS and LC admission type (elective or urgent). Further subgroups will be defined:

i. Patients referred for elective surgery versus patients undergoing urgent surgery;
ii. Patients with normal LFTs at baseline (i.e., low risk) versus patients with abnormal (outside of local Trust upper and/or lower normal limits) LFTs at baseline (i.e., moderate risk);
iii. Patients with a history of pancreatitis versus patients with no history of pancreatitis.

Health economic analysis

The primary economic evaluation will compare NHS costs and patient outcomes, measured by Quality Adjusted Life Years (QALYs), between the groups on an ITT basis. This analysis will explore whether the initial savings due to not using MRCP are offset by higher treatment costs and worse patient outcomes due to LC complications and/or retained stones.

English NHS tariffs will be used to estimate the costs.[17] These tariffs are based on Healthcare Resource Groups (HRGs) and distinguish between open/laparoscopic, elective/urgent and day case/inpatient surgery. However, they are not sufficiently granular to measure the impact on costs of small differences in theatre time or post-surgical length of stay which might be evident between groups. Therefore, micro-costing may also be used to estimate the incremental costs of LC. HRG codes, NHS tariffs and national unit costs will be used to estimate all other care costs during follow up.[18] National Institute for Health and Care Excellence recommended EQ-5D-5L value sets for

For peer review only - http://bmjopen.bmj.com/site/about/guidelines.xhtml

England will be used with linear interpolation between time-points and adjustment for baseline to calculate QALYs. [19, 20]

Costs and outcomes beyond 12 months will be discounted at standard rates.[21] Missing cost and EQ-5D-5L data will be described and multiple imputation techniques used as appropriate. The incremental cost per QALY of MRCP versus EM over the 18-month follow-up period will be estimated, and bootstrapping techniques used to estimate 95% confidence intervals. Regression analyses will be used to estimate the incremental net monetary benefit and cost-effectiveness acceptability curve of MRCP at conventional thresholds after controlling for key baseline covariates.[22] In a secondary economic analysis, we will estimate the cost per gallstone-related re-admission avoided. Sensitivity analyses will explore the robustness of the conclusions to plausible differences in key costing assumptions. If there is evidence that costs and outcome differences between groups persist between 6 and 18 months, a simple extrapolation model to estimate cost-effectiveness beyond 18 months will be considered. The impact of care pathways on patient productivity costs will be described in secondary analyses. A health economics analysis plan will detail analytical methods.

Safety

In accordance with Good Clinical Practice guidelines, the occurrence of adverse events will be monitored carefully and recorded in detail.

Following LC, transient post-operative complications are not unexpected and often delay the patient’s hospital discharge. These complications are classified as expected events in this study. There are also some known complications of ERCP and MRCP which are also classified as expected events. Any event classified as expected will not require expedited reporting to the Sponsor or Research Ethics Committee (REC), unless in the event of a participant death. Unexpected serious

For peer review only - http://bmjopen.bmj.com/site/about/guidelines.xhtml

adverse events, including participant deaths, will be reported to the study Sponsor and coordinating centre.

Data on adverse events will be collected from randomisation until hospital discharge after LC, or for 9-months post-randomisation for participants who do not undergo LC for any reason.

Patient and Public Involvement statement

We will work in close partnership with patients and members of the public throughout all phases of the Sunflower study. The study funding proposal was developed in collaboration with our patient/public contributor co-applicant who has experience of gallstone investigations/surgery. Two public contributors have been appointed to the Study Steering Committee, who participate in all committee meetings.

Feedback from patient and public contributors on study rationale and design was sought during the pre- and post-funding stages of the study. Topics discussed included experiences of gallstone investigations and bile duct imaging, advantages/disadvantages of bile duct imaging, relevance/importance of a randomised trial and barriers/facilitators to recruitment, randomisation, and follow-up to inform development of the study protocol.

A patient/public advisory group has been established, and patient and public involvement activities have been conducted regularly throughout the set-up and running of the study. Consultations with patient/public contributors have covered a diverse range of issues. For example, patient/public contributors have been involved in drafting and revising study participant information leaflets and consent forms to improve participant information provision. We have also consulted with patient/public contributors regarding proposed protocol amendments, resulting in the introduction of an additional postal/remote consent pathway to optimise recruitment during the COVID-19 pandemic. Interviews with patient/public contributors have also been conducted to explore

For peer review only

For peer review only - http://bmjopen.bmj.com/site/about/guidelines.xhtml

concerns around study participation and attending hospital during the pandemic. Most participants were in favour of recruitment restarting, helping to inform subsequent decisions around restarting the study. Further patient/public activities have been undertaken to explore additional strategies for optimising recruitment (including the co-development of a patient-facing clinic recruitment poster), patients' experiences/burden of study participation and layout and content of the Sunflower study website.

We will work closely with patient and public contributors to develop a strategy for sharing the study findings with participants, future patients, service users and members of the wider public. This will include consideration of disseminating findings using a range of materials and methods, such as plain language summaries, newsletters, videos, social media, websites, and patient support groups.

Risk of Bias

Participating centres will aim to screen all adult patients with symptomatic gallbladder disease who are scheduled and fit for LC. We expect that the recruited patients will be generalisable to the wider population. Patients allocated to each group will have comparable baseline characteristics as randomisation will take place after consent via a secure website with allocation concealment. Allocations will be stratified by centre to minimise confounding.

The primary outcome is objective and will be obtained from routinely collected data sources. This is important as participants, clinicians and other hospital staff caring for participants will be unblinded. Attempts to blind participants by undertaking "dummy" MRCP in those randomised to EM would have added significant additional research costs and created logistical issues. Because "dummy" MRCP would cause a similar delay to LC as actual MRCP, it would also prevent the study from assessing the impact of this on outcomes.

For peer review only - http://bmjopen.bmj.com/site/about/guidelines.xhtml

The risk of missing data has been minimised by using routinely collected data for the primary outcome, and by undertaking regular review of missing data captured explicitly for the study.

We do not expect patient-reported outcomes to be at risk of bias, since we do not anticipate that participants will have preferences or expectations about the use of MRCP. We aim to promote uncertainty about the effects of the two interventions by providing information that describes the risks and benefits of each intervention. This information has been reviewed by a Public and Patient Involvement group and informed by the experiences of the QRI researchers.

Study management and oversight

Preparation of study documents, site initiation visits and training, day-to-day running of the study and monitoring of centres is managed by the Clinical Trials and Evaluation Unit, Bristol Trials Centre. A Study Executive Group (SEG) is overseeing the study and meets regularly to review milestones. A Data Monitoring and Safety Committee (DMSC) meets biannually to review data. A Study Steering Committee (SSC) oversees the study and has ultimate responsibility for any decision about its continuation. Membership of the study oversight committees is described in the Acknowledgments section.

Changes to the study protocol

Two substantial amendments have been approved by the REC. The first, in April 2019, added the option of postal consent and the second, in December 2019, revised some inclusion/exclusion criteria and clarified information relating to withdrawals in line with the Health Research Authority guidance.

For peer review only - http://bmjopen.bmj.com/site/about/guidelines.xhtml

DISCUSSION

The main challenge facing the Sunflower study is the recruitment target of 13,680 participants. Recruitment started 1 month later than planned in January 2019. The initial focus was to open centres to recruitment, with a target of 36 centres recruiting within phase 1. The study has already exceeded this target. As of 23rd August 2020, 48 centres were open to recruitment and 2,040 participants had been randomised. The QRI will be key to optimising recruitment, providing training at investigators meetings and recruitment tips for staff at participating centres.[23, 24] With a 48-month recruitment period, it will also be key for the study to keep centres engaged. Initiatives include the use of an active Twitter account, regular recruitment updates and centre targeted emails, bi-annual newsletters and annual investigators' meetings. It will also be important to remain aware of other relevant research which could impact on the study. The study team will undertake annual literature reviews.

The study includes patients who are scheduled for LC as either an elective or urgent procedure. It is important to ensure that both pathways are represented to promote generalisability of the study results, but it is recognised that recruitment of urgent patients is challenging. These patients can be admitted at any time of day and over weekends when research nurses may not be available to approach patients and receive consent. As of 23rd August 2020, 77.8% of randomised participants were elective referrals and 22.2% were from urgent admissions. The study is keen to involve surgical trainees in the recruitment process, and is participating in the National Institute for Health Research (NIHR) associate PI scheme.[25] During study training, the local team is encouraged to consider recruitment from the urgent pathway, and the ratio of elective to urgent patients recruited is being monitored. The study feels that surgical trainees will be critical to the successful recruitment of urgent patients. Discussions from an investigator meeting identified that the inclusion criterion, ALT and/or ALP less than 2xULN, was disproportionally excluding urgent patients and so this was amended to 3xULN. The impact of this amendment will be monitored.

For peer review only - http://bmjopen.bmj.com/site/about/guidelines.xhtml

For the study results to be generalisable, we aim to recruit both patients classified as being at low and moderate risk of CBD stones. Low risk patients are defined as those with bilirubin, ALT and ALP all within the normal range of their local Trust. As of 23rd August 2020, 67% of randomised participants were classified as being at low risk and 33% as moderate risk.

Another major challenge will be obtaining routinely collected data from England, Wales, Northern Ireland and Scotland. Applications to obtain routinely collected data are complex and time-consuming, and a different application process is required for each nation. The coordinating centre has considerable experience in completing these applications, but they must be successful and timely for the primary outcome to be determined and health economic analyses completed.

The primary outcome for the study is composed of three elements. The first will compare hospital admissions for treatment of gallstone complications in the 18 months after randomisation. The second and third elements will compare post-operative complications of LC and complications of ERCP. These two elements are important to measure, as there is evidence of increased rates of some complications (e.g., biliary leakage) in patients with retained stones post LC.[26] This may have relevance to participants randomised to EM. It is also possible that participants are over-treated with MRCP, and any subsequent ERCP, and suffer avoidable complications. These elements will help inform study results and application in practice. The primary outcome will be reported both overall and by each individual element.

STUDY STATUS

Recruitment to the study started in January 2019 and is due to complete in January 2023. The current protocol is version 3.0 dated 25th November 2019. Relevant regulatory approvals will be obtained for amendments to the study documentation. Information relating to amendments will be disseminated to relevant parties via email, newsletter and an electronic study management system.

For peer review only - http://bmjopen.bmj.com/site/about/guidelines.xhtml

LIST OF ABBREVIATIONS

ALP: Alkaline phosphatase

ALT: Alanine transferase

CBD: Common bile duct

DMSC: Data Monitoring and Safety Committee

EM: Expectant management

ERCP: Endoscopic retrograde cholangiopancreatography

HES: Hospital Episode Statistics

HRG: Healthcare Resource Group

HRQoL: Health Related Quality of Life

ITT: Intention-to-treat

LC: Laparoscopic cholecystectomy

LFT: Liver function test

MRCP: Magnetic resonance cholangiopancreatography

NHS: National Health Service

NIHR: National Institute for Health Research

QALY: Quality Adjusted Life Year

QRI: QuinteT Recruitment Intervention

REC: Research Ethics Committee

SEG: Study Executive Group

For peer review only

For peer review only - http://bmjopen.bmj.com/site/about/guidelines.xhtml

SSC: Study Steering Committee

UK: United Kingdom

ULN: Upper limit of normal

USS: Ultrasound scan

ACKNOWLEDGEMENTS

The authors would like to thank all study staff involved in recruitment, coordination and data collection for this study, and members of the Patient and Public Involvement group who assisted with study design and review of study documents. Special thanks are also given to the clinical and nursing teams at participating centres for their support in the conduct of the study. The authors would like to acknowledge the DMSC and SSC for their oversight of the study. The independent DMSC is formed of a Chairperson, one consultant surgeon and one consultant radiologist. The SSC is formed of a Chairperson, two consultant surgeons, two consultant radiologists, one medical statistician and two public and patient involvement representatives; other SSC members with observer status represent the SEG in these committees.

The Sunflower SEG is made up of the following: Madeleine Clout, Jane Blazeby, Chris Rogers, Barnaby Reeves, Michelle Lazaroo, Kerry Avery, Natalie Blencowe, Ravi Vohra, Neil Jennings, William Hollingworth, Joanna Thorn, Marcus Jepson, Jane Collingwood, Ashley Guthrie, Elizabeth Booth, Samir Pathak, Ian Beckingham, Lucy Culliford, Ewen Griffiths, Raneem Albazaz and Giles Toogood.

Patients and/or the public were involved in the design of this research. Patients and/or the public are/will be involved in the conduct, reporting, and dissemination plans of this research. Refer to the Methods section for further details.

For peer review only

The study is formally supported by the Association of Upper Gastrointestinal Surgeons of Great Britain and Ireland and the Great Britain & Ireland Hepato Pancreato Biliary Association, both of which recognise the importance of the objectives of this study.

Ethics and dissemination

This study was reviewed and given a favourable opinion by Yorkshire & The Humber – South Yorkshire REC on the 10th December 2018 (REC reference: 18/YH/0358). Each participating centre is required to provide evidence of local confirmation of capacity and capability prior to starting recruitment to the study. Informed consent to participate will be obtained from all study participants. Participants have the right to withdraw from the study at any time and, if they do withdraw, will be treated according to standard local hospital procedures. Routine data will continue to be collected for withdrawn participants, unless the participant explicitly asks for this data collection to stop. Study registration number: ISRCTN10378861.

The study findings will be disseminated through peer-reviewed publications and international conferences, and provided to study participants. A study specific Twitter account will be used to promote the study, provide updates on milestones and disseminate study findings. After study completion and reporting, the technical appendix, statistical code and dataset generated from the study will be available from the corresponding author on request. The request must include a specification of the data requested and justification for that request (i.e., a statement of purpose for which the data are required). The data may not be released unless all Bristol Trials Centre and Sponsor requirements are fulfilled (e.g., protocol, sample size calculation, REC and Health Research Authority approval if appropriate, a collaboration arrangement in place between the Sponsor and the external body).

For peer review only

For peer review only - http://bmjopen.bmj.com/site/about/guidelines.xhtml

DECLARATIONS

Competing interests

The authors declare that they have no competing interests.

Funding statement

This work is supported by the NIHR Health Technology Assessment Programme (Grant Ref: 16/142/04). The funder had no role in the design of the study, data collection or writing this manuscript. The study is also supported by the Royal College of Surgeons Surgical Trials Centre in Bristol. JB, CR, BR and KA are all supported by the NIHR Bristol and Weston Biomedical Research Centre. JB is a NIHR Senior Investigator. The study Sponsor is Leeds Teaching Hospitals NHS Trust (Research Governance Manager Anne Gowing, anne.gowing@nhs.net) and the study is managed by the Bristol Trials Centre, Clinical Trials and Evaluation Unit. This study was designed and is being delivered in collaboration with the Bristol Trials Centre, Clinical Trials and Evaluation Unit, a UK Clinical Research Collaboration registered clinical trials unit which is in receipt of NIHR clinical trials unit support funding. The research team acknowledges the support of the NIHR Clinical Research Network. The Sunflower study is overseen by an independent SSC and DMSC. The study has been designed with input from public and patient groups.

AUTHOR CONTRIBUTIONS

MC: Preparation and drafting of study protocol, first draft and writing of manuscript.

JB: Study concept and design, preparation and review of study protocol, pre-study feasibility work, definition of study intervention and outcomes, review of manuscript and support for Chief Investigator.

For peer review only

For peer review only - http://bmjopen.bmj.com/site/about/guidelines.xhtml

CR: Study design, preparation and drafting of study protocol, sample size and statistical analysis plan, review of manuscript.

BR: Study concept and design, preparation and drafting of study protocol, review of manuscript.

ML: Statistical analyses, statistical analysis plan, review of manuscript.

KA: Study design, review of study protocol, leading PPI, review of manuscript.

NB: Study concept and design, review of study protocol, review of manuscript.

RV: Study concept and design, review of study protocol, review of manuscript.

NJ: Study concept and design, review of study protocol, review of manuscript.

WH: Study design, review of study protocol, health economic analysis plan and analyses, review of manuscript.

JT: Health economic analysis plan and analyses, review of manuscript.

MJ: Study design, review of study protocol, leading recruitment intervention, review of manuscript.

JC: Review of manuscript.

AG: Study design, review of study protocol, review of manuscript.

EB: Study design, review of study protocol, review of manuscript.

SP: Study design, review of study protocol, review of manuscript.

IB: Study concept and design, review of study protocol, review of manuscript.

LC: Study design, review of study protocol, review of manuscript.

EG: Study concept and design, review of study protocol, review of manuscript.

RA: Study concept and design, review of study protocol, review of manuscript.

For peer review only

GT: Study concept and design, preparation of study protocol, definition of study intervention and outcomes, review of manuscript. Chief investigator.

All authors read and approved the final manuscript.

For peer review only

For peer review only - http://bmjopen.bmj.com/site/about/guidelines.xhtml

REFERENCES

1. Sinha S, Hofman D, Stoker DL, et al. Epidemiological study of provision of cholecystectomy in England from 2000 to 2009: retrospective analysis of Hospital Episode Statistics. Surgical endoscopy. 2013;27(1):162-75.
2. Maple JT, Ikenberry SO, Anderson MA, et al. The role of endoscopy in the management of choledocholithiasis. Gastrointestinal endoscopy. 2011;74(4):731-44.
3. Costi R, Gnocchi A, Di Mario F, et al. Diagnosis and management of choledocholithiasis in the golden age of imaging, endoscopy and laparoscopy. World journal of gastroenterology. 2014;20(37):13382-401.
4. Treckmann J, Sauerland S, Frilling A, et al. Common Bile Duct Stones – Update 2006. In: Neugebauer EAM, Sauerland S, Fingerhut A, Millat B, Buess G, editors. EAES Guidelines for Endoscopic Surgery: Twelve Years Evidence-Based Surgery in Europe. Berlin, Heidelberg: Springer Berlin Heidelberg; 2006. p. 329-33.
5. Warttig S, Ward S, Rogers G. Diagnosis and management of gallstone disease: summary of NICE guidance. BMJ (Clinical research ed). 2014;349:g6241.
6. Williams EJ, Green J, Beckingham I, et al. Guidelines on the management of common bile duct stones (CBDS). Gut. 2008;57(7):1004-21.
7. CholeS Study Group WMRC. Population-based cohort study of outcomes following cholecystectomy for benign gallbladder diseases. Br J Surg. 2016;103(12):1704-15.
8. Noel R, Arnelo U, Lundell L, et al. Index versus delayed cholecystectomy in mild gallstone pancreatitis: results of a randomized controlled trial. HPB. 2018;20(10):932-8.
9. Anderson MA, Fisher L, Jain R, et al. Complications of ERCP. Gastrointestinal endoscopy. 2012;75(3):467-73.
10. Donovan JL, Rooshenas L, Jepson M, et al. Optimising recruitment and informed consent in randomised controlled trials: the development and implementation of the Quintet Recruitment Intervention (QRI). Trials. 2016;17(1):283.
11. Herbert A, Wijlaars L, Zylbersztejn A, et al. Data Resource Profile: Hospital Episode Statistics Admitted Patient Care (HES APC). International Journal of Epidemiology. 2017;46(4):1093-i.
12. EuroQoL Research Foundation. EQ-5D-5L User Guide: Basic information on how to use the EQ-5D-5L instrument2019 25 October 2019; 3.0. Available from: https://euroqol.org/wp-content/uploads/2019/09/EQ-5D-5L-English-User-Guide_version-3.0-Sept-2019-secured.pdf.
13. Gillies K. Development of core outcome set for symptomatic uncomplicated gallstone disease University of Aberdeen [Available from: https://www.abdn.ac.uk/hsru/what-we-do/research/projects/core-outcome-for-uncomplicated-gallstone-disease-398.php.
14. COMET Initiative. Development of core outcome set for symptomatic uncomplicated gallstone disease [Available from: http://www.comet-initiative.org/studies/details/927?result=true.
15. CONSORT Group. CONSORT Transparent Reporting of Trials [Available from: http://www.consort-statement.org/.
16. D'Agostino RB, Sr., Massaro JM, Sullivan LM. Non-inferiority trials: design concepts and issues - the encounters of academic consultants in statistics. Stat Med. 2003;22(2):169-86.
17. NHS England. National Tariff [Available from: https://www.england.nhs.uk/resources/pay-syst/national-tariff/.
18. Curtis LB, A. Unit Costs of Health and Social Care 2018. University of Kent; 2018 13 December 2018.
19. NICE. Position statement on use of the EQ-5D-5L value set for England (updated October 2019) 2019 [updated October 2019. Available from: https://www.nice.org.uk/about/what-we-do/our-programmes/nice-guidance/technology-appraisal-guidance/eq-5d-5l.
20. Manca A, Hawkins N, Sculpher MJ. Estimating mean QALYs in trial-based cost-effectiveness analysis: the importance of controlling for baseline utility. Health Econ. 2005;14(5):487-96.
21. Excellence NIfHaC. Guide to the methods of technology appraisal 2013 2013 [Available from: https://www.nice.org.uk/process/pmg9/chapter/foreword.

For peer review only - http://bmjopen.bmj.com/site/about/guidelines.xhtml

22. Hoch JS, Briggs AH, Willan AR. Something old, something new, something borrowed, something blue: a framework for the marriage of health econometrics and cost-effectiveness analysis. Health Econ. 2002;11(5):415-30.

23. Mills N, Blazeby JM, Hamdy FC, et al. Training recruiters to randomized trials to facilitate recruitment and informed consent by exploring patients' treatment preferences. Trials. 2014;15(1):323.

24. Paramasivan S, Rogers CA, Welbourn R, et al. Enabling recruitment success in bariatric surgical trials: pilot phase of the By-Band-Sleeve study. International Journal of Obesity. 2017;41(11):1654-61.

25. NIHR - National Institute for Health Research. Associate Principal Investigator (PI) Scheme 2019 [updated 30/05/2019. Available from: https://www.nihr.ac.uk/documents/associate-principal-investigator-pi-scheme/11694?diaryentryid=53625.

26. Z'Graggen K, Aronsky D, Maurer CA, et al. Acute postoperative pancreatitis after laparoscopic cholecystectomy. Results of the Prospective Swiss Association of Laparoscopic and Thoracoscopic Surgery Study. Archives of surgery (Chicago, Ill : 1960). 1997;132(9):1026-30; discussion 31.

LEGENDS

Figure 1

LC = Laparoscopic cholecystectomy; MRCP = Magnetic resonance cholangiopancreatography

Table 1

[a] These timepoints are months post randomisation.

[b] During phase 1 of the study, some recruitment consultations will be audio-recorded at high volume centres with patient consent. Thereafter, some consultations will be recorded at centres where recruitment rates fall below target.

[c] Participants will not be asked to complete the laparoscopic cholecystectomy admission questionnaires if they have completed the baseline questionnaires within the previous two days.

For peer review only - http://bmjopen.bmj.com/site/about/guidelines.xhtml

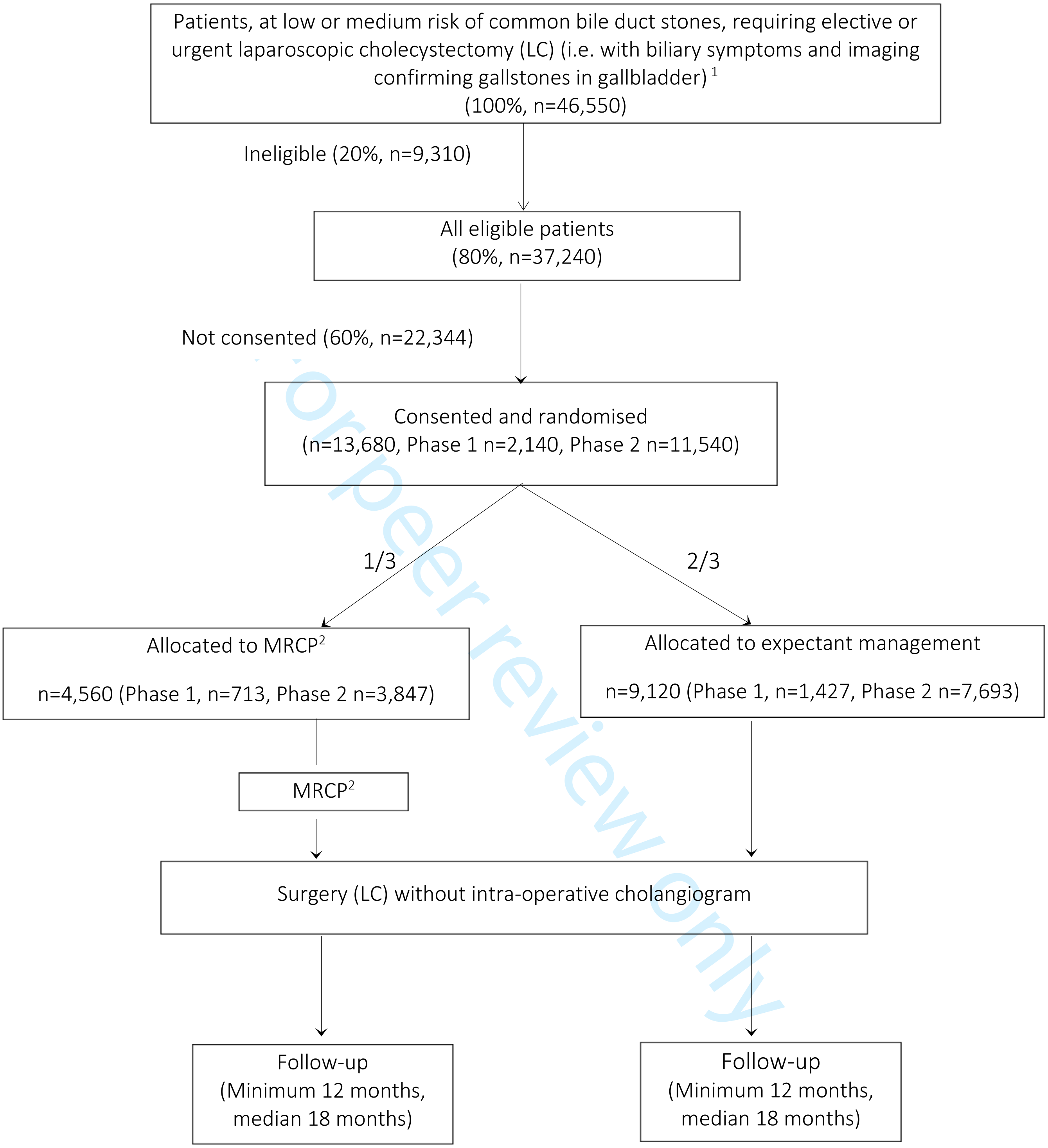

For peer review only

Reporting checklist for protocol of a clinical trial.

Based on the SPIRIT guidelines.

Instructions to authors

Complete this checklist by entering the page numbers from your manuscript where readers will find each of the items listed below.

Your article may not currently address all the items on the checklist. Please modify your text to include the missing information. If you are certain that an item does not apply, please write "n/a" and provide a short explanation.

Upload your completed checklist as an extra file when you submit to a journal.

In your methods section, say that you used the SPIRITreporting guidelines, and cite them as:

Chan A-W, Tetzlaff JM, Altman DG, Laupacis A, Gøtzsche PC, Krleža-Jerić K, Hróbjartsson A, Mann H, Dickersin K, Berlin J, Doré C, Parulekar W, Summerskill W, Groves T, Schulz K, Sox H, Rockhold FW, Rennie D, Moher D. SPIRIT 2013 Statement: Defining standard protocol items for clinical trials. Ann Intern Med. 2013;158(3):200-207

For peer review only

		Reporting Item	Page Number
**Administrative information**			
Title	#1	Descriptive title identifying the study design, population, interventions, and, if applicable, trial acronym	1
Trial registration	#2a	Trial identifier and registry name. If not yet registered, name of intended registry	6, 25
Trial registration: data set	#2b	All items from the World Health Organization Trial Registration Data Set	n/a, not a registry trial
Protocol version	#3	Date and version identifier	23
Funding	#4	Sources and types of financial, material, and other support	26
Roles and	#5a	Names, affiliations, and roles of protocol contributors	1-3

responsibilities: contributorship			
Roles and responsibilities: sponsor contact information	#5b	Name and contact information for the trial sponsor	26
Roles and responsibilities: sponsor and funder	#5c	Role of study sponsor and funders, if any, in study design; collection, management, analysis, and interpretation of data; writing of the report; and the decision to submit the report for publication, including whether they will have ultimate authority over any of these activities	26
Roles and responsibilities: committees	#5d	Composition, roles, and responsibilities of the coordinating centre, steering committee, endpoint adjudication committee, data management team, and other individuals or groups overseeing the trial, if applicable (see Item 21a for data monitoring committee)	24, 25
**Introduction**			
Background and rationale	#6a	Description of research question and justification for undertaking the trial, including summary of relevant studies (published and unpublished) examining benefits and harms for each intervention	8, 9
Background and rationale: choice of comparators	#6b	Explanation for choice of comparators	8, 9
Objectives	#7	Specific objectives or hypotheses	9
Trial design	#8	Description of trial design including type of trial (eg, parallel group, crossover, factorial, single group), allocation ratio, and framework (eg, superiority, equivalence, non-inferiority, exploratory)	10
**Methods: Participants, interventions, and outcomes**			

For peer review only

Study setting	#9	Description of study settings (eg, community clinic, academic hospital) and list of countries where data will be collected. Reference to where list of study sites can be obtained	10
Eligibility criteria	#10	Inclusion and exclusion criteria for participants. If applicable, eligibility criteria for study centres and individuals who will perform the interventions (eg, surgeons, psychotherapists)	10, 11
Interventions: description	#11a	Interventions for each group with sufficient detail to allow replication, including how and when they will be administered	15
Interventions: modifications	#11b	Criteria for discontinuing or modifying allocated interventions for a given trial participant (eg, drug dose change in response to harms, participant request, or improving / worsening disease)	25
Interventions: adherance	#11c	Strategies to improve adherence to intervention protocols, and any procedures for monitoring adherence (eg, drug tablet return; laboratory tests)	10
Interventions: concomitant care	#11d	Relevant concomitant care and interventions that are permitted or prohibited during the trial	10, 11
Outcomes	#12	Primary, secondary, and other outcomes, including the specific measurement variable (eg, systolic blood pressure), analysis metric (eg, change from baseline, final value, time to event), method of aggregation (eg, median, proportion), and time point for each outcome. Explanation of the clinical relevance of chosen efficacy and harm outcomes is strongly recommended	11-13
Participant timeline	#13	Time schedule of enrolment, interventions (including any run-ins and washouts), assessments, and visits for participants. A schematic diagram is highly recommended (see Figure)	13
Sample size	#14	Estimated number of participants needed to achieve study objectives and how it was determined, including clinical and statistical assumptions supporting any	14

For peer review only

		sample size calculations	
Recruitment	#15	Strategies for achieving adequate participant enrolment to reach target sample size	14
**Methods: Assignment of interventions (for controlled trials)**			
Allocation: sequence generation	#16a	Method of generating the allocation sequence (eg, computer-generated random numbers), and list of any factors for stratification. To reduce predictability of a random sequence, details of any planned restriction (eg, blocking) should be provided in a separate document that is unavailable to those who enrol participants or assign interventions	15
Allocation concealment mechanism	#16b	Mechanism of implementing the allocation sequence (eg, central telephone; sequentially numbered, opaque, sealed envelopes), describing any steps to conceal the sequence until interventions are assigned	15
Allocation: implementation	#16c	Who will generate the allocation sequence, who will enrol participants, and who will assign participants to interventions	15
Blinding (masking)	#17a	Who will be blinded after assignment to interventions (eg, trial participants, care providers, outcome assessors, data analysts), and how	20
Blinding (masking): emergency unblinding	#17b	If blinded, circumstances under which unblinding is permissible, and procedure for revealing a participant's allocated intervention during the trial	n/a, no blinding
**Methods: Data collection, management, and analysis**			
Data collection plan	#18a	Plans for assessment and collection of outcome, baseline, and other trial data, including any related processes to promote data quality (eg, duplicate	16

For peer review only

		measurements, training of assessors) and a description of study instruments (eg, questionnaires, laboratory tests) along with their reliability and validity, if known. Reference to where data collection forms can be found, if not in the protocol	
Data collection plan: retention	#18b	Plans to promote participant retention and complete follow-up, including list of any outcome data to be collected for participants who discontinue or deviate from intervention protocols	25
Data management	#19	Plans for data entry, coding, security, and storage, including any related processes to promote data quality (eg, double data entry; range checks for data values). Reference to where details of data management procedures can be found, if not in the protocol	16
Statistics: outcomes	#20a	Statistical methods for analysing primary and secondary outcomes. Reference to where other details of the statistical analysis plan can be found, if not in the protocol	17
Statistics: additional analyses	#20b	Methods for any additional analyses (eg, subgroup and adjusted analyses)	17-19
Statistics: analysis population and missing data	#20c	Definition of analysis population relating to protocol non-adherence (eg, as randomised analysis), and any statistical methods to handle missing data (eg, multiple imputation)	17-19
**Methods: Monitoring**			
Data monitoring: formal committee	#21a	Composition of data monitoring committee (DMC); summary of its role and reporting structure; statement of whether it is independent from the sponsor and competing interests; and reference to where further details about its charter can be found, if not in the protocol. Alternatively, an explanation of why a DMC is not needed	24
Data monitoring:	#21b	Description of any interim analyses and stopping	17

For peer review only

interim analysis		guidelines, including who will have access to these interim results and make the final decision to terminate the trial	
Harms	#22	Plans for collecting, assessing, reporting, and managing solicited and spontaneously reported adverse events and other unintended effects of trial interventions or trial conduct	19, 20
Auditing	#23	Frequency and procedures for auditing trial conduct, if any, and whether the process will be independent from investigators and the sponsor	15
**Ethics and dissemination**			
Research ethics approval	#24	Plans for seeking research ethics committee / institutional review board (REC / IRB) approval	25
Protocol amendments	#25	Plans for communicating important protocol modifications (eg, changes to eligibility criteria, outcomes, analyses) to relevant parties (eg, investigators, REC / IRBs, trial participants, trial registries, journals, regulators)	23
Consent or assent	#26a	Who will obtain informed consent or assent from potential trial participants or authorised surrogates, and how (see Item 32)	14
Consent or assent: ancillary studies	#26b	Additional consent provisions for collection and use of participant data and biological specimens in ancillary studies, if applicable	n/a
Confidentiality	#27	How personal information about potential and enrolled participants will be collected, shared, and maintained in order to protect confidentiality before, during, and after the trial	16
Declaration of interests	#28	Financial and other competing interests for principal investigators for the overall trial and each study site	26
Data access	#29	Statement of who will have access to the final trial dataset, and disclosure of contractual agreements that	25, 26

For peer review only

		limit such access for investigators	
Ancillary and post trial care	#30	Provisions, if any, for ancillary and post-trial care, and for compensation to those who suffer harm from trial participation	Not included
Dissemination policy: trial results	#31a	Plans for investigators and sponsor to communicate trial results to participants, healthcare professionals, the public, and other relevant groups (eg, via publication, reporting in results databases, or other data sharing arrangements), including any publication restrictions	25
Dissemination policy: authorship	#31b	Authorship eligibility guidelines and any intended use of professional writers	26-28
Dissemination policy: reproducible research	#31c	Plans, if any, for granting public access to the full protocol, participant-level dataset, and statistical code	25
**Appendices**			
Informed consent materials	#32	Model consent form and other related documentation given to participants and authorised surrogates	Available on request
Biological specimens	#33	Plans for collection, laboratory evaluation, and storage of biological specimens for genetic or molecular analysis in the current trial and for future use in ancillary studies, if applicable	n/a, no specimens

None The SPIRIT checklist is distributed under the terms of the Creative Commons Attribution License CC-BY-ND 3.0. This checklist can be completed online using https://www.goodreports.org/, a tool made by the EQUATOR Network in collaboration with Penelope.ai

For peer review only

BMJ Open

Study protocol for a randomised controlled trial to establish the clinical and cost effectiveness of expectant management versus pre-operative imaging with magnetic resonance cholangiopancreatography in patients with symptomatic gallbladder disease undergoing laparoscopic cholecystectomy at low or moderate risk of common bile duct stones (The Sunflower Study)

Journal:	*BMJ Open*
Manuscript ID	bmjopen-2020-044281.R2
Article Type:	Protocol
Date Submitted by the Author:	20-Mar-2021
Complete List of Authors:	Clout, Madeleine; University of Bristol Faculty of Medical and Veterinary Sciences, Clinical Trials and Evaluation Unit<br>Blazeby, Jane; University of Bristol, Department of Social Medicine<br>Rogers, Chris; university of bristol, Clinical Science South Bristol<br>Reeves, Barnaby; University of Bristol, Bristol Heart Institute<br>Lazaroo, Michelle; University of Bristol Faculty of Medical and Veterinary Sciences, Clinical Trials and Evaluation Unit<br>Avery, Kerry; University of Bristol, School of Social and Community Medicine<br>Blencowe, Natalie; University of Bristol, Centre for Surgical Research, School of Social and Community Medicine<br>Vohra, Ravi; Nottingham City Hospital<br>Jennings, Neil; Sunderland Royal Hospital<br>Hollingworth, William; University of Bristol, School of Social and Community Medicine<br>Thorn, Joanna; University of Bristol, School of Social and Community Medicine<br>Jepson, Marcus; University of Bristol School of Social and Community Medicine,<br>Collingwood, Jane; University of Bristol, School of Population Health Sciences<br>Guthrie, Ashley; Leeds Teaching Hospitals NHS Trust<br>Booth, Elizabeth; NHS Coventry and Rugby Clinical Commissioning Group<br>Pathak, Samir; University of Bristol, Centre for Surgical Research, Population Health Sciences<br>Beckingham, Ian; Nottingham University Hospitals NHS Trust<br>Culliford, Lucy; University of Bristol, Bristol Heart Institute,<br>Griffiths, Ewen; University Hospitals Birmingham NHS Foundation Trust, Department of Upper GI Surgery<br>Albazaz, Raneem; Leeds Teaching Hospitals NHS Trust<br>Toogood, Giles; Leeds Teaching Hospitals NHS Trust
**Primary Subject Heading**:	Surgery

Secondary Subject Heading:	Gastroenterology and hepatology, Radiology and imaging, Health economics, Qualitative research
Keywords:	Magnetic resonance imaging < RADIOLOGY & IMAGING, Endoscopy < GASTROENTEROLOGY, SURGERY, GASTROENTEROLOGY, NEPHROLOGY, Clinical trials < THERAPEUTICS

SCHOLARONE™
Manuscripts

I, the Submitting Author has the right to grant and does grant on behalf of all authors of the Work (as defined in the below author licence), an exclusive licence and/or a non-exclusive licence for contributions from authors who are: i) UK Crown employees; ii) where BMJ has agreed a CC-BY licence shall apply, and/or iii) in accordance with the terms applicable for US Federal Government officers or employees acting as part of their official duties; on a worldwide, perpetual, irrevocable, royalty-free basis to BMJ Publishing Group Ltd ("BMJ") its licensees and where the relevant Journal is co-owned by BMJ to the co-owners of the Journal, to publish the Work in this journal and any other BMJ products and to exploit all rights, as set out in our licence.

The Submitting Author accepts and understands that any supply made under these terms is made by BMJ to the Submitting Author unless you are acting as an employee on behalf of your employer or a postgraduate student of an affiliated institution which is paying any applicable article publishing charge ("APC") for Open Access articles. Where the Submitting Author wishes to make the Work available on an Open Access basis (and intends to pay the relevant APC), the terms of reuse of such Open Access shall be governed by a Creative Commons licence – details of these licences and which Creative Commons licence will apply to this Work are set out in our licence referred to above.

Other than as permitted in any relevant BMJ Author's Self Archiving Policies, I confirm this Work has not been accepted for publication elsewhere, is not being considered for publication elsewhere and does not duplicate material already published. I confirm all authors consent to publication of this Work and authorise the granting of this licence.

For peer review only

Study protocol for a randomised controlled trial to establish the clinical and cost effectiveness of expectant management versus pre-operative imaging with magnetic resonance cholangiopancreatography in patients with symptomatic gallbladder disease undergoing laparoscopic cholecystectomy at low or moderate risk of common bile duct stones (The Sunflower Study)

On behalf of the Sunflower Study Executive Group*

*Authors for 'Sunflower Study Executive Group':

1. Madeleine Clout, Clinical Trials and Evaluation Unit, Bristol Trials Centre, University of Bristol, Bristol Royal Infirmary, Zone A Level 7, Bristol, BS2 8HW, UK, madeleine.clout@bristol.ac.uk
2. Jane Blazeby, NIHR Bristol Biomedical Research Centre, Centre for Surgical Research, Bristol Medical School, Population Health Sciences, University of Bristol, Office 4.05 Canynge Hall, 39 Whatley Road, Bristol, BS8 2PS, UK, j.m.blazeby@bristol.ac.uk
3. Chris Rogers, Clinical Trials and Evaluation Unit, Bristol Trials Centre, University of Bristol, Bristol Royal Infirmary, Zone A Level 7, Bristol, BS2 8HW, UK, chris.rogers@bristol.ac.uk
4. Barnaby C Reeves, Clinical Trials and Evaluation Unit, Bristol Trials Centre, University of Bristol, Bristol Royal Infirmary, Zone A Level 7, Bristol, BS2 8HW, UK, barney.reeves@bristol.ac.uk
5. Michelle Lazaroo, Clinical Trials and Evaluation Unit, Bristol Trials Centre, University of Bristol, Bristol Royal Infirmary, Zone A Level 7, Bristol, BS2 8HW, UK, michelle.lazaroo@bristol.ac.uk
6. Kerry Avery, NIHR Bristol Biomedical Research Centre and Centre for Surgical Research Bristol Medical School, Population Health Sciences, University of Bristol, 39 Whatley Road, Bristol, BS8 2PS, UK, Kerry.Avery@bristol.ac.uk

For peer review only

7. Natalie Blencowe, NIHR Bristol Biomedical Research Centre and Centre for Surgical Research, Population Health Sciences, Bristol Medical School, University of Bristol, 39 Whatley Road, Bristol, BS8 2PS, UK, Natalie.Blencowe@bristol.ac.uk
8. Ravi Vohra, Trent Oesophago-Gastric Unit, Nottingham University Hospitals NHS Trust, City Hospital, Hucknall Rd, Nottingham, NG5 1PB, UK, ravinder.vohra@nuh.nhs.com
9. Neil Jennings, Sunderland Royal Hospital, Kayll Road, Sunderland, Tyne & Wear, SR4 7TP, UK, Neil.Jennings@chsft.nhs.uk
10. William Hollingworth, Bristol Medical School, Population Health Sciences, University of Bristol, Office 2.08, 1-5 Whiteladies Road, Bristol, BS8 1NU, UK, william.hollingworth@bristol.ac.uk
11. Joanna Thorn, Bristol Medical School, Population Health Sciences, University of Bristol, Office 2.12, 1-5 Whiteladies Road, Bristol, BS8 1NU, UK, joanna.thorn@bristol.ac.uk
12. Marcus Jepson, Bristol Medical School, Population Health Sciences, University of Bristol, Office 4.03 Canynge Hall, 39 Whatley Road, Bristol, BS8 2PS, UK, marcus.jepson@bristol.ac.uk
13. Jane Collingwood, Bristol Medical School, Population Health Sciences, University of Bristol, Office 2.01 Canynge Hall, 39 Whatley Road, Bristol, BS8 2PS, UK, jane.collingwood@bristol.ac.uk
14. Ashley Guthrie, Leeds Teaching Hospitals NHS Trust, St James's University Hospital, Beckett Street, Leeds, LS9 7TF, UK, ashley.guthrie@nhs.net
15. Elizabeth Booth, NHS Coventry and Rugby Clinical Commissioning Group, Parkside House, Quinton Road, Coventry, CV1 2NJ, UK, elizabeth.booth@coventryrugbyccg.nhs.uk
16. Samir Pathak, Centre for Surgical Research and Centre for Surgical Research, Population Health Sciences, Bristol Medical School, University of Bristol, 39 Whatley Road, Bristol, BS8 2PS, UK, drsamirpathak@gmail.com

For peer review only

For peer review only - http://bmjopen.bmj.com/site/about/guidelines.xhtml

17. Ian Beckingham, Nottingham University Hospitals NHS Trust, Hucknall Road, Nottingham, NG5 1PB, UK, ian.beckingham@nuh.nhs.uk
18. Lucy Culliford, Clinical Trials & Evaluation Unit, Bristol Trials Centre, University of Bristol, Bristol Royal Infirmary, Zone A Level 7, Bristol, BS2 8HW, UK, lucy.culliford@bristol.ac.uk
19. Ewen A Griffiths, University Hospitals Birmingham NHS Foundation Trust, Queen Elizabeth Hospital Birmingham, Mindelsohn Way, Edgbaston, Birmingham, B15 2GW, UK, Ewen.Griffiths@uhb.nhs.uk
20. Raneem Albazaz, Leeds Teaching Hospitals NHS Trust, St James's University Hospital, Beckett Street, Leeds, LS9 7TF, UK, r.albazaz@nhs.net
21. Giles Toogood, Department of Hepatobiliary and Transplantation Surgery, Leeds Teaching Hospitals NHS Trust, St James's University Hospital, Beckett Street, Leeds, LS9 7TF, UK, giles.toogood@nhs.net

Corresponding author

Mrs Madeleine Clout

madeleine.clout@bristol.ac.uk

Clinical Trials and Evaluation Unit

Bristol Trials Centre

University of Bristol

Bristol Royal Infirmary, Zone A Level 7

Bristol

BS2 8HW

0117 342 2526 / 07929 771395

For peer review only

For peer review only - http://bmjopen.bmj.com/site/about/guidelines.xhtml

Word count: 4516

Keywords: Gallstones, gallbladder disease, laparoscopic cholecystectomy, magnetic resonance cholangiopancreatography, randomised controlled trial, common bile duct, endoscopic retrograde cholangiopancreatography

For peer review only

ABSTRACT:

Introduction

Surgery to remove the gallbladder (laparoscopic cholecystectomy (LC)) is the standard treatment for symptomatic gallbladder disease. One potential complication of gallbladder disease is that gallstones can pass into the common bile duct (CBD) where they may remain dormant, pass spontaneously into the bowel or cause problems such as obstructive jaundice or pancreatitis. Patients requiring LC are assessed pre-operatively for their risk of CBD stones using liver function tests and imaging. If the risk is high, guidelines recommend further investigation and treatment. Further investigation of patients at low or moderate risk of CBD stones is not standardised, and the practice of imaging the CBD using magnetic resonance cholangiopancreatography (MRCP) in these patients varies across the United Kingdom (UK). The consequences of these decisions may lead to over or under treatment of patients.

Methods and analysis

We are conducting a UK multicentre, pragmatic, open, randomised controlled trial with internal pilot phase to compare the effectiveness and cost-effectiveness of pre-operative imaging with MRCP versus expectant management (i.e. no pre-operative imaging) in adult patients with symptomatic gallbladder disease undergoing urgent or elective LC who are at low or moderate risk of CBD stones. We aim to recruit 13,680 patients over 48 months. The primary outcome is any hospital admission within 18 months of randomisation for a complication of gallstones. This includes complications of endoscopic retrograde cholangiopancreatography for the treatment of gallstones, and complications of LC. This will be determined using routine data sources, e.g. NHS Digital Hospital Episode Statistics for participants in England. Secondary outcomes include cost-effectiveness and patient-reported quality of life, with participants followed up for a median of 18 months.

Ethics and dissemination

For peer review only - http://bmjopen.bmj.com/site/about/guidelines.xhtml

This study received approval from Yorkshire & The Humber – South Yorkshire Research Ethics Committee. Results will be submitted for publication in a peer-reviewed journal.

Registration details: ISRCTN10378861

For peer review only - http://bmjopen.bmj.com/site/about/guidelines.xhtml

ARTICLE SUMMARY

Strengths and limitations of this study

- This is a randomised controlled trial with randomisation taking place using a secure internet-based system to ensure allocation concealment, which will ensure that patients allocated to each group have comparable baseline characteristics.
- The study includes an internal pilot phase with progression to the main study dependent on a number of criteria, to ensure that the study will only proceed if it is viable to do so.
- Participants, clinicians and other hospital staff caring for participants will not be blind to the study allocation.
- The criteria used to define low and moderate risk of common bile duct stones have been identified by clinicians within the study team, as there is no universal agreed definition of these terms.
- The study primary outcome will be derived from routine data sources, which should ensure high levels of data completeness but is reliant on timely approval of numerous applications to holders of routine data.

For peer review only

INTRODUCTION

Surgery to remove the gallbladder, known as laparoscopic cholecystectomy (LC), is the standard treatment for symptomatic gallbladder disease. LC is one of the most common operations undertaken globally and around 70,000 LC operations are performed annually in England.[1]

One potential complication of gallbladder disease is that gallstones may pass from the gallbladder into the common bile duct (CBD). Once in the CBD, they may remain dormant, pass spontaneously into the bowel or cause problems such as obstructive jaundice, cholangitis or pancreatitis. If CBD stones are found, current guidelines recommend removal either before or during LC. Therefore, patients requiring LC are assessed pre-operatively for their risk of CBD stones using a combination of liver function tests (LFTs) and imaging. If the risk is high, guidelines recommend further investigation and treatment.[2-6] Further investigation of patients at moderate or low risk of CBD stones is not standardised and practice across the United Kingdom (UK) varies. Fewer than 10% of these patients actually have CBD stones.[2-4, 6, 7] Consequently, surgeons make different decisions about investigation of the CBD; some surgeons choose to perform diagnostic imaging tests for these patients, and others do not. This variation likely results in over or under treatment, with significant risks to the patient in terms of morbidity, Health Related Quality of Life (HRQoL) and costs to the health service.

A UK-wide audit found that a third of patients undergoing LC, irrespective of risk of CBD stones, had diagnostic imaging to test for CBD stones.[7] This is most frequently performed with magnetic resonance cholangiopancreatography (MRCP) before admission for LC, however some surgeons instead use intra-operative cholangiogram and other intra-operative ultrasound examinations of the CBD. MRCP produces detailed images of the biliary and pancreatic ducts, involves a 1-hour hospital visit and costs the National Health Service (NHS) about £365. MRCP can identify CBD stones but may delay LC, which can have a detrimental effect on HRQoL and can lead to increased problems with gallstones or a more complex LC.[8] If a CBD stone is identified during MRCP, extraction is most

For peer review only - http://bmjopen.bmj.com/site/about/guidelines.xhtml

frequently performed before LC with an endoscopic retrograde cholangiopancreatography (ERCP). The risks and mortality associated with ERCP are significant,[9] as is the cost to the NHS (about £1,600). Not having MRCP avoids these risks but can lead to CBD stones remaining after surgery, which may also cause complications. Research is needed to establish whether it is appropriate for patients at low and moderate risk of CBD stones to proceed straight to LC without imaging the CBD with MRCP.

METHODS AND ANALYSIS

Aims and Objectives

The Sunflower study aims to compare the effectiveness and cost-effectiveness of pre-operative imaging with MRCP versus no MRCP (hereafter called expectant management (EM)) in patients with symptomatic gallbladder disease undergoing LC who are at low or moderate risk of CBD stones. The study will test the hypothesis that EM is not inferior to MRCP with respect to hospitalisation for treatment for a complication of gallstones up to 18 months after randomisation.

The objectives are to estimate the:

1. Difference between groups in the proportion of participants requiring a hospital admission for treatment of a complication of gallstones in the gallbladder or CBD.
2. Difference between groups with respect to a range of secondary outcomes, including patient-reported HRQoL, symptoms related to complications of gallstones in the gallbladder or CBD, and symptoms related to complications associated with LC and, where applicable, ERCP.
3. Cost-effectiveness of MRCP compared with EM.

For peer review only - http://bmjopen.bmj.com/site/about/guidelines.xhtml

Study Design

The Sunflower study is a multi-centre, pragmatic, open, randomised controlled trial with an internal pilot. The study has an embedded Qualitative Research Integrated within Trials Recruitment Intervention (QRI) to optimise informed consent and recruitment.[10]

There are two phases to the study:

Phase 1 (internal pilot): Set-up and recruit from at least 36 centres by month 16 of recruitment, with integrated QRI to optimise recruitment.

Phase 2: Increase the number of centres to at least 50 and continue recruitment using the methods established in Phase 1, along with integrated QRI to optimise recruitment and promote adherence in new centres.

Progression to phase 2 is conditional on the study demonstrating to the funder that: a) at least 30 centres are open and recruiting; b) at least 1,750 participants have been randomised; c) at least 90% of participants have followed their allocated pathway; and d) the primary outcome can be identified reliably from routine data.

Setting

Recruitment will take place at a minimum of 50 NHS hospitals across the UK with a recruitment target of 13,680 participants over a 48-month period (

Figure **1**).

Study Population

The target population is adults (18 years or older) with symptomatic gallbladder disease, scheduled and fit for LC as an elective or urgent procedure, who are at low or moderate risk of CBD stones. Low

For peer review only

For peer review only - http://bmjopen.bmj.com/site/about/guidelines.xhtml

or moderate risk is defined as CBD diameter ≤8mm on ultrasound scan (USS), and bilirubin ≤50umol/l, and alanine transferase (ALT) less than three times the upper limit of normal (≤3xULN) and/or alkaline phosphatase (ALP) ≤3xULN. If the CBD cannot be visualised on USS, the patient may be recruited if no intrahepatic duct dilatation is reported.

Patients are excluded if they are unable to undergo MRCP, have evidence of empyema or perforated gallbladder requiring urgent intervention, have had a previous gastric bypass, have had a previous MRCP or endoscopic USS within the last 3 months, have had any previous ERCP or have haemolytic disease. Patients must be willing to participate in follow up and be able to provide informed consent.

Figure 1: The study schema

Primary Outcome

The primary outcome is any of the following:

i. Any hospital admission within 18 months of randomisation for treatment of a complication of gallstones whether in the CBD or gallbladder;
ii. Complications during the admission for LC for the treatment for gallstones or any readmission for complications of the LC leading to a hospital day of >2 days. Complications will include, but not be limited to, return to theatre post LC for any cause, percutaneous radiological drainage and ERCP for non-diagnostic reasons (e.g. for a bile leak). It does not include a diagnostic ERCP performed following an MRCP where CBD stones were identified;
iii. Complications during any ERCP for the treatment for gallstones. Complications will include, but are not limited to, blood transfusion post ERCP, percutaneous radiological drainage, treatment of a perforation occurring during ERCP, acute pancreatitis and other complications leading to a hospital stay of > 2 days.

For peer review only - http://bmjopen.bmj.com/site/about/guidelines.xhtml

This outcome will be ascertained by linking the study data with routinely-collected Hospital Episode Statistics (HES) and mortality data from NHS Digital for participants recruited in England, and the equivalent data sources for participants recruited in Scotland, Wales and Northern Ireland. Routine data sources contain information on care provided to all patients treated in UK NHS hospitals.[11] The final specification of qualifying events for identifying the primary outcome from these routine sources will be developed and validated during phase 1 of the study, through comparison of routine data with events identified from review of the medical records at 90 days post-LC.

Secondary Outcomes

Patient-reported HRQoL will be measured using the EQ-5D-5L questionnaire.[12] To minimise the overall study burden, about 20% of participants will be sampled and asked to complete questionnaires at multiple timepoints (Table 1). A questionnaire pack will be provided to participants containing the validated EQ-5D-5L questionnaire plus questions about the impact of abdominal pain on work, productivity and primary healthcare use.

For peer review only

For peer review only - http://bmjopen.bmj.com/site/about/guidelines.xhtml

Data item	Pre-Randomisation	Pre-Surgery	Hospital admission for LC	90 days post LC	3 -months[a]	6-months[a]	12-months[a]	18-months[a]
Eligibility	✓							
Written informed consent	✓							
Medical history	✓							
EQ-5D-5L, productivity & primary care use questionnaire (20% sample)	✓		✓[c]		✓	✓	✓	✓
MRCP, ERCP and intraoperative imaging details, if applicable		✓						
Operative and post-operative details			✓					
Items in the symptomatic gallstone core outcome set				✓				
Safety data				✓				
Study consultations audio recorded for QRI [b]	✓							

Table 1: Data collection time points

Other secondary outcomes include items that cover the domains in the core outcome set for patients with symptomatic gallstones, and hospital resource use in the 18 months after randomisation collected from routine data sources.[13, 14]

For peer review only

For peer review only - http://bmjopen.bmj.com/site/about/guidelines.xhtml

Sample Size

The sample size of 13,680 has been chosen to test the hypothesis that EM is non-inferior to MRCP with respect to the primary outcome. In estimating the sample size, we have considered the proportion of patients that would be expected to experience the primary outcome, as identified in the CholeS audit (5% to 10%) and through exploration of HES data.[7] This includes patients at high risk of CBD stones. The consensus amongst clinicians on the study team was that the non-inferiority margin should be set at 1.5%, i.e. that the risk of the primary outcome with EM should not exceed 8.5% assuming a risk of 7% after MRCP. A study of 13,680 participants will provide 90% power to test the non-inferiority hypothesis for a 7% event risk with MRCP and 80% power to test the non-inferiority hypothesis for a higher event risk of 8.5%., assuming 2.5% one-sided statistical significance and a 1:2 allocation ratio (MRCP:EM). The 20% sample of participants providing HRQoL data will be stratified by centre. This sample of 2,736 participants will have >90% power to detect a small effect size of 0.12 standard deviations between groups on the EQ-5D-5L questionnaire.

Recruitment

Recruiting centres will screen LC waiting lists and gallbladder clinic lists for potentially eligible patients. Potential participants will also be identified from urgent inpatient and ambulatory care admissions and ward handovers. Eligible patients will be provided with study information and will have the opportunity to ask questions before being approached for their informed consent (see Supplemental Material 1). Patients who feel they have had insufficient time to consider the study will be invited to take home a consent form and questionnaire pack (where applicable), and to complete and return them by post. Details of all patients approached for the study and reasons for non-participation will be documented.

For peer review only - http://bmjopen.bmj.com/site/about/guidelines.xhtml

Randomisation

Randomisation will be performed by an authorised member of the local research team using a secure internet-based system to ensure allocation concealment. The allocation will be computer generated and stratified by centre.

Participants will be randomised to MRCP or EM in a 1:2 ratio. This ratio has been selected to reflect current levels of MRCP provision (13-26% of patients at low or moderate risk of CBD stones currently undergo MRCP).[7]

Interventions

The study interventions are pre-operative MRCP and EM.

Participants allocated to the MRCP group will have an MRCP before the date of their listed LC. The study will make no changes to the usual hospital radiology protocols used for MRCP and will not impose any timelines for the MRCP, or any subsequent interventions. If a CBD stone is found on MRCP, surgeons can manage extraction according to their usual practice. The study does not stipulate removal of identified CBD stones, and any further interventions are at the discretion of the clinician. If an ERCP is requested, it will be performed as a separate inpatient procedure before admission for LC.

Participants allocated to EM will be listed for LC without any pre-operative imaging. If MRCP is carried out, e.g. due to a change in a participant's clinical circumstances after randomisation, this will be documented as a crossover.

In both groups, intraoperative imaging will not be permitted unless there is an anatomical reason to do so, or if a CBD stone is found on MRCP. Any deviations from the protocol will be recorded and monitored.

For peer review only

Data collection

Data will be collected by the local study team at baseline, before, during and after LC, and at hospital discharge (Table 1). Data collection will be from hospital records, including operative notes, and will include information on any pre-LC MRCP and ERCP, and any intra-operative imaging. Data will either be collected onto a paper case report form and transcribed into a secure electronic study database or entered directly into the database. During phase 1, safety data will also be collected from hospital records 90 days post-LC by the local study team.

Linkage to routine data

With consent, participant identifiers will be sent to NHS Digital, and equivalent organisations for participants recruited outside of England. These organisations will match and extract pseudonymised data for these participants for all episodes of hospital care for a minimum of 18 months from the point of randomisation, and for a period of 12 months prior to randomisation. The data will include inpatient and critical care episodes, accident and emergency department attendances, out-patient appointments, diagnostic imaging investigations and mortality.

MRCP Quality Assurance

To address the challenge of MRCP technique varying across participating centres, images and reports for a random 10% sample of participants who receive an MRCP will be transferred for independent quality assurance review by consultant radiologists. The MRCP examinations will be assessed for the type of sequences performed, the diagnostic quality of the images and level of concordance with the hospital reports. This data will provide an overview of current NHS practice and the overall quality of MRCP examinations, as well as characterising variation in MRCP technique in study centres.

For peer review only - http://bmjopen.bmj.com/site/about/guidelines.xhtml

313 **Statistical analysis**

314 The study will be analysed on an intention-to-treat (ITT) basis, i.e. outcomes will be analysed
315 according to the allocated treatment pathway irrespective of future management and events, and
316 every effort will be made to include all randomised participants. Non-adherence to the treatment
317 allocation will be documented. The primary analysis will follow the Consolidated Standards of
318 Reporting Trials guidelines for a non-inferiority study.[15] An analysis according to treatment
319 pathway followed will also be performed for the primary outcome. As recommended, both analyses
320 will be considered when assessing whether the hypothesis is met.[16] The primary outcome will be
321 compared using survival methods to allow for censoring. For participants without a qualifying
322 primary outcome event, they will be censored at the time the dataset was compiled. If more than
323 one qualifying event occurs (e.g. two hospital admissions for gallstone-related complications), time
324 to the first event will be used. The frequency of each element of the primary outcome and reasons
325 for admission, where applicable, will be described. Secondary outcomes will be compared using a
326 mixed model for continuous outcomes or a generalised linear regression model for binary outcomes,
327 adjusted for pre-randomisation measures when available. For secondary outcomes measured at
328 multiple time points, changes in treatment effect with time since randomisation will be assessed by
329 adding a treatment by time interaction to the model and comparing models using a likelihood ratio
330 test. Model fit will be assessed and alternative models and/or transformations (e.g. to induce
331 normality) will be explored where appropriate. Sensitivity analyses using multiple imputation for
332 missing data will be explored. Analyses will be adjusted for centre and treatment differences will be
333 reported with 95% confidence intervals.

334 A detailed analysis plan will be prepared. There is no intention to compare any outcomes between
335 groups at the end of phase 1; only descriptive statistics about eligibility, recruitment and adherence
336 will be summarised at this time to determine whether the study satisfies the progression criteria.

337 Exploratory analyses will include:

For peer review only

For peer review only - http://bmjopen.bmj.com/site/about/guidelines.xhtml

i. Relationship between number and size of stones seen on MRCP and patient outcome (cohort undergoing MRCP only);
ii. Relationship between stones removed under ERCP or not (e.g., 'necessary' vs. 'unnecessary' ERCP) and patient outcomes (cohort undergoing ERCP only).

Subgroup analyses will evaluate the primary outcome in patients defined by characteristics. The main subgroup analysis will be in patients with low versus moderate risk of CBD stones, which will be defined following phase 1. This definition is expected to be a composite of baseline LFTs, baseline CBD diameter on USS and LC admission type (elective or urgent). Further subgroups will be defined:

i. Patients referred for elective surgery versus patients undergoing urgent surgery;
ii. Patients with normal LFTs at baseline (i.e., low risk) versus patients with abnormal (outside of local Trust upper and/or lower normal limits) LFTs at baseline (i.e., moderate risk);
iii. Patients with a history of pancreatitis versus patients with no history of pancreatitis.

Health economic analysis

The primary economic evaluation will compare NHS costs and patient outcomes, measured by Quality Adjusted Life Years (QALYs), between the groups on an ITT basis. This analysis will explore whether the initial savings due to not using MRCP are offset by higher treatment costs and worse patient outcomes due to LC complications and/or retained stones.

English NHS tariffs will be used to estimate the costs.[17] These tariffs are based on Healthcare Resource Groups (HRGs) and distinguish between open/laparoscopic, elective/urgent and day case/inpatient surgery. However, they are not sufficiently granular to measure the impact on costs of small differences in theatre time or post-surgical length of stay which might be evident between groups. Therefore, micro-costing may also be used to estimate the incremental costs of LC. HRG codes, NHS tariffs and national unit costs will be used to estimate all other care costs during follow up.[18] National Institute for Health and Care Excellence recommended EQ-5D-5L value sets for

For peer review only - http://bmjopen.bmj.com/site/about/guidelines.xhtml

England will be used with linear interpolation between time-points and adjustment for baseline to calculate QALYs. [19, 20]

Costs and outcomes beyond 12 months will be discounted at standard rates.[21] Missing cost and EQ-5D-5L data will be described and multiple imputation techniques used as appropriate. The incremental cost per QALY of MRCP versus EM over the 18-month follow-up period will be estimated, and bootstrapping techniques used to estimate 95% confidence intervals. Regression analyses will be used to estimate the incremental net monetary benefit and cost-effectiveness acceptability curve of MRCP at conventional thresholds after controlling for key baseline covariates.[22] In a secondary economic analysis, we will estimate the cost per gallstone-related re-admission avoided. Sensitivity analyses will explore the robustness of the conclusions to plausible differences in key costing assumptions. If there is evidence that costs and outcome differences between groups persist between 6 and 18 months, a simple extrapolation model to estimate cost-effectiveness beyond 18 months will be considered. The impact of care pathways on patient productivity costs will be described in secondary analyses. A health economics analysis plan will detail analytical methods.

Safety

In accordance with Good Clinical Practice guidelines, the occurrence of adverse events will be monitored carefully and recorded in detail.

Following LC, transient post-operative complications are not unexpected and often delay the patient's hospital discharge. These complications are classified as expected events in this study. There are also some known complications of ERCP and MRCP which are also classified as expected events. Any event classified as expected will not require expedited reporting to the Sponsor or Research Ethics Committee (REC), unless in the event of a participant death. Unexpected serious

For peer review only

adverse events, including participant deaths, will be reported to the study Sponsor and coordinating centre.

Data on adverse events will be collected from randomisation until hospital discharge after LC, or for 9-months post-randomisation for participants who do not undergo LC for any reason.

Patient and Public Involvement statement

We will work in close partnership with patients and members of the public throughout all phases of the Sunflower study. The study funding proposal was developed in collaboration with our patient/public contributor co-applicant who has experience of gallstone investigations/surgery. Two public contributors have been appointed to the Study Steering Committee, who participate in all committee meetings.

Feedback from patient and public contributors on study rationale and design was sought during the pre- and post-funding stages of the study. Topics discussed included experiences of gallstone investigations and bile duct imaging, advantages/disadvantages of bile duct imaging, relevance/importance of a randomised trial and barriers/facilitators to recruitment, randomisation, and follow-up to inform development of the study protocol.

A patient/public advisory group has been established, and patient and public involvement activities have been conducted regularly throughout the set-up and running of the study. Consultations with patient/public contributors have covered a diverse range of issues. For example, patient/public contributors have been involved in drafting and revising study participant information leaflets and consent forms to improve participant information provision. We have also consulted with patient/public contributors regarding proposed protocol amendments, resulting in the introduction of an additional postal/remote consent pathway to optimise recruitment during the COVID-19 pandemic. Interviews with patient/public contributors have also been conducted to explore

For peer review only

concerns around study participation and attending hospital during the pandemic. Most participants were in favour of recruitment restarting, helping to inform subsequent decisions around restarting the study. Further patient/public activities have been undertaken to explore additional strategies for optimising recruitment (including the co-development of a patient-facing clinic recruitment poster), patients' experiences/burden of study participation and layout and content of the Sunflower study website.

We will work closely with patient and public contributors to develop a strategy for sharing the study findings with participants, future patients, service users and members of the wider public. This will include consideration of disseminating findings using a range of materials and methods, such as plain language summaries, newsletters, videos, social media, websites, and patient support groups.

Risk of Bias

Participating centres will aim to screen all adult patients with symptomatic gallbladder disease who are scheduled and fit for LC. We expect that the recruited patients will be generalisable to the wider population. Patients allocated to each group will have comparable baseline characteristics as randomisation will take place after consent via a secure website with allocation concealment. Allocations will be stratified by centre to minimise confounding.

The primary outcome is objective and will be obtained from routinely collected data sources. This is important as participants, clinicians and other hospital staff caring for participants will be unblinded. Attempts to blind participants by undertaking "dummy" MRCP in those randomised to EM would have added significant additional research costs and created logistical issues. Because "dummy" MRCP would cause a similar delay to LC as actual MRCP, it would also prevent the study from assessing the impact of this on outcomes.

For peer review only - http://bmjopen.bmj.com/site/about/guidelines.xhtml

The risk of missing data has been minimised by using routinely collected data for the primary outcome, and by undertaking regular review of missing data captured explicitly for the study.

We do not expect patient-reported outcomes to be at risk of bias, since we do not anticipate that participants will have preferences or expectations about the use of MRCP. We aim to promote uncertainty about the effects of the two interventions by providing information that describes the risks and benefits of each intervention. This information has been reviewed by a Public and Patient Involvement group and informed by the experiences of the QRI researchers.

Study management and oversight

Preparation of study documents, site initiation visits and training, day-to-day running of the study and monitoring of centres is managed by the Clinical Trials and Evaluation Unit, Bristol Trials Centre. A Study Executive Group (SEG) is overseeing the study and meets regularly to review milestones. A Data Monitoring and Safety Committee (DMSC) meets biannually to review data. A Study Steering Committee (SSC) oversees the study and has ultimate responsibility for any decision about its continuation. Membership of the study oversight committees is described in the Acknowledgments section.

Changes to the study protocol

Two substantial amendments have been approved by the REC. The first, in April 2019, added the option of postal consent and the second, in December 2019, revised some inclusion/exclusion criteria and clarified information relating to withdrawals in line with the Health Research Authority guidance.

For peer review only - http://bmjopen.bmj.com/site/about/guidelines.xhtml

DISCUSSION

The main challenge facing the Sunflower study is the recruitment target of 13,680 participants. Recruitment started 1 month later than planned in January 2019. The initial focus was to open centres to recruitment, with a target of 36 centres recruiting within phase 1. The study has already exceeded this target. As of 23rd August 2020, 48 centres were open to recruitment and 2,040 participants had been randomised. The QRI will be key to optimising recruitment, providing training at investigators meetings and recruitment tips for staff at participating centres.[23, 24] With a 48-month recruitment period, it will also be key for the study to keep centres engaged. Initiatives include the use of an active Twitter account, regular recruitment updates and centre targeted emails, bi-annual newsletters and annual investigators' meetings. It will also be important to remain aware of other relevant research which could impact on the study. The study team will undertake annual literature reviews.

The study includes patients who are scheduled for LC as either an elective or urgent procedure. It is important to ensure that both pathways are represented to promote generalisability of the study results, but it is recognised that recruitment of urgent patients is challenging. These patients can be admitted at any time of day and over weekends when research nurses may not be available to approach patients and receive consent. As of 23rd August 2020, 77.8% of randomised participants were elective referrals and 22.2% were from urgent admissions. The study is keen to involve surgical trainees in the recruitment process, and is participating in the National Institute for Health Research (NIHR) associate PI scheme.[25] During study training, the local team is encouraged to consider recruitment from the urgent pathway, and the ratio of elective to urgent patients recruited is being monitored. The study feels that surgical trainees will be critical to the successful recruitment of urgent patients. Discussions from an investigator meeting identified that the inclusion criterion, ALT and/or ALP less than 2xULN, was disproportionally excluding urgent patients and so this was amended to 3xULN. The impact of this amendment will be monitored.

For peer review only - http://bmjopen.bmj.com/site/about/guidelines.xhtml

For the study results to be generalisable, we aim to recruit both patients classified as being at low and moderate risk of CBD stones. Low risk patients are defined as those with bilirubin, ALT and ALP all within the normal range of their local Trust. As of 23rd August 2020, 67% of randomised participants were classified as being at low risk and 33% as moderate risk.

Another major challenge will be obtaining routinely collected data from England, Wales, Northern Ireland and Scotland. Applications to obtain routinely collected data are complex and time-consuming, and a different application process is required for each nation. The coordinating centre has considerable experience in completing these applications, but they must be successful and timely for the primary outcome to be determined and health economic analyses completed.

The primary outcome for the study is composed of three elements. The first will compare hospital admissions for treatment of gallstone complications in the 18 months after randomisation. The second and third elements will compare post-operative complications of LC and complications of ERCP. These two elements are important to measure, as there is evidence of increased rates of some complications (e.g., biliary leakage) in patients with retained stones post LC.[26] This may have relevance to participants randomised to EM. It is also possible that participants are over-treated with MRCP, and any subsequent ERCP, and suffer avoidable complications. These elements will help inform study results and application in practice. The primary outcome will be reported both overall and by each individual element.

STUDY STATUS

Recruitment to the study started in January 2019 and is due to complete in January 2023. The current protocol is version 3.0 dated 25th November 2019. Relevant regulatory approvals will be obtained for amendments to the study documentation. Information relating to amendments will be disseminated to relevant parties via email, newsletter and an electronic study management system.

For peer review only - http://bmjopen.bmj.com/site/about/guidelines.xhtml

Ethics and dissemination

This study was reviewed and given a favourable opinion by Yorkshire & The Humber – South Yorkshire REC on the 10th December 2018 (REC reference: 18/YH/0358). Each participating centre is required to provide evidence of local confirmation of capacity and capability prior to starting recruitment to the study. Informed consent to participate will be obtained from all study participants. Participants have the right to withdraw from the study at any time and, if they do withdraw, will be treated according to standard local hospital procedures. Routine data will continue to be collected for withdrawn participants, unless the participant explicitly asks for this data collection to stop. Study registration number: ISRCTN10378861.

The study findings will be disseminated through peer-reviewed publications and international conferences, and provided to study participants. A study specific Twitter account will be used to promote the study, provide updates on milestones and disseminate study findings. After study completion and reporting, the technical appendix, statistical code and dataset generated from the study will be available from the corresponding author on request. The request must include a specification of the data requested and justification for that request (i.e., a statement of purpose for which the data are required). The data may not be released unless all Bristol Trials Centre and Sponsor requirements are fulfilled (e.g., protocol, sample size calculation, REC and Health Research Authority approval if appropriate, a collaboration arrangement in place between the Sponsor and the external body).

LIST OF ABBREVIATIONS

ALP: Alkaline phosphatase

ALT: Alanine transferase

CBD: Common bile duct

For peer review only

DMSC: Data Monitoring and Safety Committee

EM: Expectant management

ERCP: Endoscopic retrograde cholangiopancreatography

HES: Hospital Episode Statistics

HRG: Healthcare Resource Group

HRQoL: Health Related Quality of Life

ITT: Intention-to-treat

LC: Laparoscopic cholecystectomy

LFT: Liver function test

MRCP: Magnetic resonance cholangiopancreatography

NHS: National Health Service

NIHR: National Institute for Health Research

QALY: Quality Adjusted Life Year

QRI: QuinteT Recruitment Intervention

REC: Research Ethics Committee

SEG: Study Executive Group

SSC: Study Steering Committee

UK: United Kingdom

ULN: Upper limit of normal

USS: Ultrasound scan

For peer review only

ACKNOWLEDGEMENTS

The authors would like to thank all study staff involved in recruitment, coordination and data collection for this study, and members of the Patient and Public Involvement group who assisted with study design and review of study documents. Special thanks are also given to the clinical and nursing teams at participating centres for their support in the conduct of the study. The authors would like to acknowledge the DMSC and SSC for their oversight of the study. The independent DMSC is formed of a Chairperson, one consultant surgeon and one consultant radiologist. The SSC is formed of a Chairperson, two consultant surgeons, two consultant radiologists, one medical statistician and two public and patient involvement representatives; other SSC members with observer status represent the SEG in these committees.

The Sunflower SEG is made up of the following: Madeleine Clout, Jane Blazeby, Chris Rogers, Barnaby Reeves, Michelle Lazaroo, Kerry Avery, Natalie Blencowe, Ravi Vohra, Neil Jennings, William Hollingworth, Joanna Thorn, Marcus Jepson, Jane Collingwood, Ashley Guthrie, Elizabeth Booth, Samir Pathak, Ian Beckingham, Lucy Culliford, Ewen Griffiths, Raneem Albazaz and Giles Toogood.

Patients and/or the public were involved in the design of this research. Patients and/or the public are/will be involved in the conduct, reporting, and dissemination plans of this research. Refer to the Methods section for further details.

The study is formally supported by the Association of Upper Gastrointestinal Surgeons of Great Britain and Ireland and the Great Britain & Ireland Hepato Pancreato Biliary Association, both of which recognise the importance of the objectives of this study.

DECLARATIONS

For peer review only - http://bmjopen.bmj.com/site/about/guidelines.xhtml

Competing interests

The authors declare that they have no competing interests.

Funding statement

This work is supported by the NIHR Health Technology Assessment Programme (Grant Ref: 16/142/04). The funder had no role in the design of the study, data collection or writing this manuscript. The study is also supported by the Royal College of Surgeons Surgical Trials Centre in Bristol. JB, CR, BR and KA are all supported by the NIHR Bristol and Weston Biomedical Research Centre. JB is a NIHR Senior Investigator. The study Sponsor is Leeds Teaching Hospitals NHS Trust (Research Governance Manager Anne Gowing, anne.gowing@nhs.net) and the study is managed by the Bristol Trials Centre, Clinical Trials and Evaluation Unit. This study was designed and is being delivered in collaboration with the Bristol Trials Centre, Clinical Trials and Evaluation Unit, a UK Clinical Research Collaboration registered clinical trials unit which is in receipt of NIHR clinical trials unit support funding. The research team acknowledges the support of the NIHR Clinical Research Network. The Sunflower study is overseen by an independent SSC and DMSC. The study has been designed with input from public and patient groups.

AUTHOR CONTRIBUTIONS

MC: Preparation and drafting of study protocol, first draft and writing of manuscript.

JB: Study concept and design, preparation and review of study protocol, pre-study feasibility work, definition of study intervention and outcomes, review of manuscript and support for Chief Investigator.

CR: Study design, preparation and drafting of study protocol, sample size and statistical analysis plan, review of manuscript.

For peer review only

BR: Study concept and design, preparation and drafting of study protocol, review of manuscript.

ML: Statistical analyses, statistical analysis plan, review of manuscript.

KA: Study design, review of study protocol, leading PPI, review of manuscript.

NB: Study concept and design, review of study protocol, review of manuscript.

RV: Study concept and design, review of study protocol, review of manuscript.

NJ: Study concept and design, review of study protocol, review of manuscript.

WH: Study design, review of study protocol, health economic analysis plan and analyses, review of manuscript.

JT: Health economic analysis plan and analyses, review of manuscript.

MJ: Study design, review of study protocol, leading recruitment intervention, review of manuscript.

JC: Review of manuscript.

AG: Study design, review of study protocol, review of manuscript.

EB: Study design, review of study protocol, review of manuscript.

SP: Study design, review of study protocol, review of manuscript.

IB: Study concept and design, review of study protocol, review of manuscript.

LC: Study design, review of study protocol, review of manuscript.

EG: Study concept and design, review of study protocol, review of manuscript.

RA: Study concept and design, review of study protocol, review of manuscript.

GT: Study concept and design, preparation of study protocol, definition of study intervention and outcomes, review of manuscript. Chief investigator.

All authors read and approved the final manuscript.

For peer review only

For peer review only - http://bmjopen.bmj.com/site/about/guidelines.xhtml

REFERENCES

1. Sinha S, Hofman D, Stoker DL, et al. Epidemiological study of provision of cholecystectomy in England from 2000 to 2009: retrospective analysis of Hospital Episode Statistics. Surgical endoscopy. 2013;27(1):162-75.

2. Maple JT, Ikenberry SO, Anderson MA, et al. The role of endoscopy in the management of choledocholithiasis. Gastrointestinal endoscopy. 2011;74(4):731-44.

3. Costi R, Gnocchi A, Di Mario F, et al. Diagnosis and management of choledocholithiasis in the golden age of imaging, endoscopy and laparoscopy. World journal of gastroenterology. 2014;20(37):13382-401.

4. Treckmann J, Sauerland S, Frilling A, et al. Common Bile Duct Stones – Update 2006. In: Neugebauer EAM, Sauerland S, Fingerhut A, Millat B, Buess G, editors. EAES Guidelines for Endoscopic Surgery: Twelve Years Evidence-Based Surgery in Europe. Berlin, Heidelberg: Springer Berlin Heidelberg; 2006. p. 329-33.

5. Warttig S, Ward S, Rogers G. Diagnosis and management of gallstone disease: summary of NICE guidance. BMJ (Clinical research ed). 2014;349:g6241.

6. Williams EJ, Green J, Beckingham I, et al. Guidelines on the management of common bile duct stones (CBDS). Gut. 2008;57(7):1004-21.

7. CholeS Study Group WMRC. Population-based cohort study of outcomes following cholecystectomy for benign gallbladder diseases. Br J Surg. 2016;103(12):1704-15.

8. Noel R, Arnelo U, Lundell L, et al. Index versus delayed cholecystectomy in mild gallstone pancreatitis: results of a randomized controlled trial. HPB. 2018;20(10):932-8.

9. Anderson MA, Fisher L, Jain R, et al. Complications of ERCP. Gastrointestinal endoscopy. 2012;75(3):467-73.

10. Donovan JL, Rooshenas L, Jepson M, et al. Optimising recruitment and informed consent in randomised controlled trials: the development and implementation of the Quintet Recruitment Intervention (QRI). Trials. 2016;17(1):283.

11. Herbert A, Wijlaars L, Zylbersztejn A, et al. Data Resource Profile: Hospital Episode Statistics Admitted Patient Care (HES APC). International Journal of Epidemiology. 2017;46(4):1093-i.

12. EuroQoL Research Foundation. EQ-5D-5L User Guide: Basic information on how to use the EQ-5D-5L instrument2019 25 October 2019; 3.0. Available from: https://euroqol.org/wp-content/uploads/2019/09/EQ-5D-5L-English-User-Guide_version-3.0-Sept-2019-secured.pdf.

13. Gillies K. Development of core outcome set for symptomatic uncomplicated gallstone disease University of Aberdeen [Available from: https://www.abdn.ac.uk/hsru/what-we-do/research/projects/core-outcome-for-uncomplicated-gallstone-disease-398.php.

14. COMET Initiative. Development of core outcome set for symptomatic uncomplicated gallstone disease [Available from: http://www.comet-initiative.org/studies/details/927?result=true.

15. CONSORT Group. CONSORT Transparent Reporting of Trials [Available from: http://www.consort-statement.org/.

16. D'Agostino RB, Sr., Massaro JM, Sullivan LM. Non-inferiority trials: design concepts and issues - the encounters of academic consultants in statistics. Stat Med. 2003;22(2):169-86.

17. NHS England. National Tariff [Available from: https://www.england.nhs.uk/resources/pay-syst/national-tariff/.

18. Curtis LB, A. Unit Costs of Health and Social Care 2018. University of Kent; 2018 13 December 2018.

19. NICE. Position statement on use of the EQ-5D-5L value set for England (updated October 2019) 2019 [updated October 2019. Available from: https://www.nice.org.uk/about/what-we-do/our-programmes/nice-guidance/technology-appraisal-guidance/eq-5d-5l.

20. Manca A, Hawkins N, Sculpher MJ. Estimating mean QALYs in trial-based cost-effectiveness analysis: the importance of controlling for baseline utility. Health Econ. 2005;14(5):487-96.

21. Excellence NIfHaC. Guide to the methods of technology appraisal 2013 2013 [Available from: https://www.nice.org.uk/process/pmg9/chapter/foreword.

For peer review only

For peer review only - http://bmjopen.bmj.com/site/about/guidelines.xhtml

22. Hoch JS, Briggs AH, Willan AR. Something old, something new, something borrowed, something blue: a framework for the marriage of health econometrics and cost-effectiveness analysis. Health Econ. 2002;11(5):415-30.
23. Mills N, Blazeby JM, Hamdy FC, et al. Training recruiters to randomized trials to facilitate recruitment and informed consent by exploring patients' treatment preferences. Trials. 2014;15(1):323.
24. Paramasivan S, Rogers CA, Welbourn R, et al. Enabling recruitment success in bariatric surgical trials: pilot phase of the By-Band-Sleeve study. International Journal of Obesity. 2017;41(11):1654-61.
25. NIHR - National Institute for Health Research. Associate Principal Investigator (PI) Scheme 2019 [updated 30/05/2019. Available from: https://www.nihr.ac.uk/documents/associate-principal-investigator-pi-scheme/11694?diaryentryid=53625.
26. Z'Graggen K, Aronsky D, Maurer CA, et al. Acute postoperative pancreatitis after laparoscopic cholecystectomy. Results of the Prospective Swiss Association of Laparoscopic and Thoracoscopic Surgery Study. Archives of surgery (Chicago, Ill : 1960). 1997;132(9):1026-30; discussion 31.

For peer review only

LEGENDS

Figure 1

LC = Laparoscopic cholecystectomy; MRCP = Magnetic resonance cholangiopancreatography

Table 1

[a] These timepoints are months post randomisation.

[b] During phase 1 of the study, some recruitment consultations will be audio-recorded at high volume centres with patient consent. Thereafter, some consultations will be recorded at centres where recruitment rates fall below target.

[c] Participants will not be asked to complete the laparoscopic cholecystectomy admission questionnaires if they have completed the baseline questionnaires within the previous two days.

For peer review only - http://bmjopen.bmj.com/site/about/guidelines.xhtml

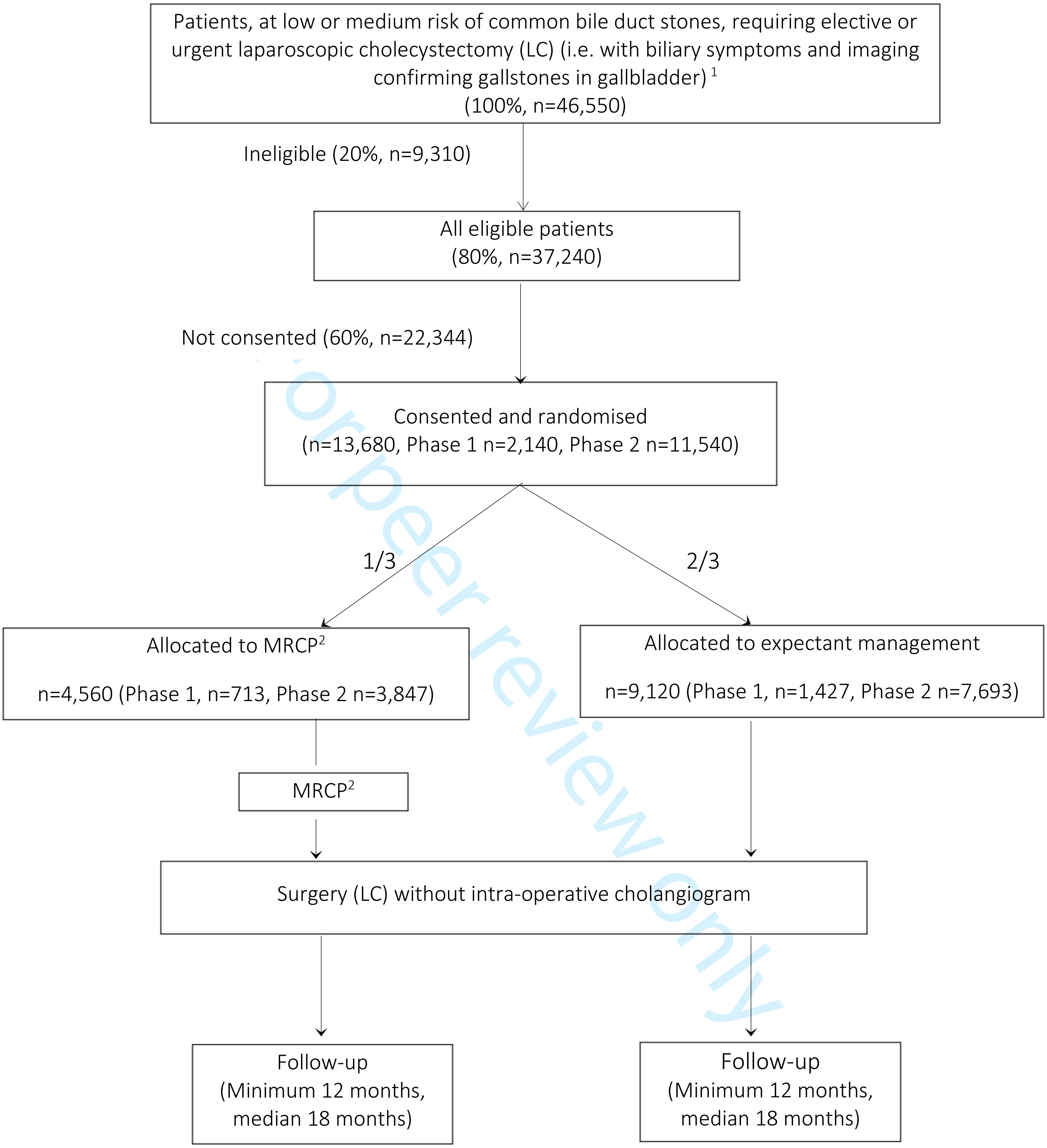

For peer review only

Patient Study ID [ ]

Place Trust logo and

PI details sticker here

HTA reference: 16/142/04
IRAS Project ID: 242342

The Sunflower Study

Testing for bile duct stones before gallbladder surgery

Participant Consent Form

Please ask the patient to complete the following: ***Initial***

1. I have read and understood the Patient Information Leaflet (dated ____/____/____, version _____). [ ]
2. I have had the opportunity to ask questions about the study and received satisfactory answers to my questions. [ ]
3. I understand that I am free to withdraw from the study at any time without giving a reason. I understand that a decision to withdraw will not affect my medical care or legal rights. [ ]
4. I give permission for information about me to be collected and shared with the Sunflower Study researchers, researchers from the University of Bristol, University Hospitals Bristol NHS Foundation Trust and Leeds Teaching Hospitals NHS Trust. This will include information collected specifically for the study and information collected as part of my routine care. I understand that strict confidentiality will be maintained at all times. [ ]
5. I give permission for my date of birth, full name, NHS/CHI number, gender and post code to be sent to <<insert text e.g. NHS Digital, PEDW>> and providers of mortality data. I understand that data held about me on these systems including hospital episodes, diagnostic imaging and mortality data will be sent to the Sunflower Study researchers at University Hospitals Bristol NHS Foundation Trust. [ ]
6. I agree to my GP being informed that I have agreed to take part in this study and to them providing relevant information from my records. [ ]
7. I understand that my MRCP scan images may be sent to a central study team at Leeds Teaching Hospitals NHS Trust for quality assurance purposes. Scans will be transferred securely using the NHS Image Exchange Portal. I understand that strict confidentiality will be maintained at all times. [ ]
8. I agree to take part in this study. [ ]

Optional section – if you do not agree to any of the following you can still take part in Sunflower ***Patient to tick Yes/No and initial***

	Yes	***No***	***Initial***
9. I agree to my information being stored securely for potential use in future ethically approved research.			

Name of patient	Signature	Date
Name of person taking consent	Signature	Date

Funded & supported by **NIHR**

1 copy for patient; 1 for research team (original); 1 to be kept with hospital notes.

The Sunflower Study is funded by the NIHR HTA Programme (project number 16/142/04). The views expressed are those of the author(s) and not necessarily those of the NHS, the NIHR or the Department of Health.

For peer review only - http://bmjopen.bmj.com/site/about/guidelines.xhtml

Reporting checklist for protocol of a clinical trial.

Based on the SPIRIT guidelines.

Instructions to authors

Complete this checklist by entering the page numbers from your manuscript where readers will find each of the items listed below.

Your article may not currently address all the items on the checklist. Please modify your text to include the missing information. If you are certain that an item does not apply, please write "n/a" and provide a short explanation.

Upload your completed checklist as an extra file when you submit to a journal.

In your methods section, say that you used the SPIRITreporting guidelines, and cite them as:

Chan A-W, Tetzlaff JM, Altman DG, Laupacis A, Gøtzsche PC, Krleža-Jerić K, Hróbjartsson A, Mann H, Dickersin K, Berlin J, Doré C, Parulekar W, Summerskill W, Groves T, Schulz K, Sox H, Rockhold FW, Rennie D, Moher D. SPIRIT 2013 Statement: Defining standard protocol items for clinical trials. Ann Intern Med. 2013;158(3):200-207

		Reporting Item	Page Number
**Administrative information**			
Title	#1	Descriptive title identifying the study design, population, interventions, and, if applicable, trial acronym	1
Trial registration	#2a	Trial identifier and registry name. If not yet registered, name of intended registry	6, 25
Trial registration: data set	#2b	All items from the World Health Organization Trial Registration Data Set	n/a, not a registry trial
Protocol version	#3	Date and version identifier	23
Funding	#4	Sources and types of financial, material, and other support	26
Roles and	#5a	Names, affiliations, and roles of protocol contributors	1-3

For peer review only

responsibilities: contributorship			
Roles and responsibilities: sponsor contact information	#5b	Name and contact information for the trial sponsor	26
Roles and responsibilities: sponsor and funder	#5c	Role of study sponsor and funders, if any, in study design; collection, management, analysis, and interpretation of data; writing of the report; and the decision to submit the report for publication, including whether they will have ultimate authority over any of these activities	26
Roles and responsibilities: committees	#5d	Composition, roles, and responsibilities of the coordinating centre, steering committee, endpoint adjudication committee, data management team, and other individuals or groups overseeing the trial, if applicable (see Item 21a for data monitoring committee)	24, 25
**Introduction**			
Background and rationale	#6a	Description of research question and justification for undertaking the trial, including summary of relevant studies (published and unpublished) examining benefits and harms for each intervention	8, 9
Background and rationale: choice of comparators	#6b	Explanation for choice of comparators	8, 9
Objectives	#7	Specific objectives or hypotheses	9
Trial design	#8	Description of trial design including type of trial (eg, parallel group, crossover, factorial, single group), allocation ratio, and framework (eg, superiority, equivalence, non-inferiority, exploratory)	10
**Methods: Participants, interventions, and outcomes**			

For peer review only

Study setting	#9	Description of study settings (eg, community clinic, academic hospital) and list of countries where data will be collected. Reference to where list of study sites can be obtained	10
Eligibility criteria	#10	Inclusion and exclusion criteria for participants. If applicable, eligibility criteria for study centres and individuals who will perform the interventions (eg, surgeons, psychotherapists)	10, 11
Interventions: description	#11a	Interventions for each group with sufficient detail to allow replication, including how and when they will be administered	15
Interventions: modifications	#11b	Criteria for discontinuing or modifying allocated interventions for a given trial participant (eg, drug dose change in response to harms, participant request, or improving / worsening disease)	25
Interventions: adherence	#11c	Strategies to improve adherence to intervention protocols, and any procedures for monitoring adherence (eg, drug tablet return; laboratory tests)	10
Interventions: concomitant care	#11d	Relevant concomitant care and interventions that are permitted or prohibited during the trial	10, 11
Outcomes	#12	Primary, secondary, and other outcomes, including the specific measurement variable (eg, systolic blood pressure), analysis metric (eg, change from baseline, final value, time to event), method of aggregation (eg, median, proportion), and time point for each outcome. Explanation of the clinical relevance of chosen efficacy and harm outcomes is strongly recommended	11-13
Participant timeline	#13	Time schedule of enrolment, interventions (including any run-ins and washouts), assessments, and visits for participants. A schematic diagram is highly recommended (see Figure)	13
Sample size	#14	Estimated number of participants needed to achieve study objectives and how it was determined, including clinical and statistical assumptions supporting any	14

For peer review only

		sample size calculations	
Recruitment	#15	Strategies for achieving adequate participant enrolment to reach target sample size	14
**Methods: Assignment of interventions (for controlled trials)**			
Allocation: sequence generation	#16a	Method of generating the allocation sequence (eg, computer-generated random numbers), and list of any factors for stratification. To reduce predictability of a random sequence, details of any planned restriction (eg, blocking) should be provided in a separate document that is unavailable to those who enrol participants or assign interventions	15
Allocation concealment mechanism	#16b	Mechanism of implementing the allocation sequence (eg, central telephone; sequentially numbered, opaque, sealed envelopes), describing any steps to conceal the sequence until interventions are assigned	15
Allocation: implementation	#16c	Who will generate the allocation sequence, who will enrol participants, and who will assign participants to interventions	15
Blinding (masking)	#17a	Who will be blinded after assignment to interventions (eg, trial participants, care providers, outcome assessors, data analysts), and how	20
Blinding (masking): emergency unblinding	#17b	If blinded, circumstances under which unblinding is permissible, and procedure for revealing a participant's allocated intervention during the trial	n/a, no blinding
**Methods: Data collection, management, and analysis**			
Data collection plan	#18a	Plans for assessment and collection of outcome, baseline, and other trial data, including any related processes to promote data quality (eg, duplicate	16

For peer review only

		measurements, training of assessors) and a description of study instruments (eg, questionnaires, laboratory tests) along with their reliability and validity, if known. Reference to where data collection forms can be found, if not in the protocol	
Data collection plan: retention	#18b	Plans to promote participant retention and complete follow-up, including list of any outcome data to be collected for participants who discontinue or deviate from intervention protocols	25
Data management	#19	Plans for data entry, coding, security, and storage, including any related processes to promote data quality (eg, double data entry; range checks for data values). Reference to where details of data management procedures can be found, if not in the protocol	16
Statistics: outcomes	#20a	Statistical methods for analysing primary and secondary outcomes. Reference to where other details of the statistical analysis plan can be found, if not in the protocol	17
Statistics: additional analyses	#20b	Methods for any additional analyses (eg, subgroup and adjusted analyses)	17-19
Statistics: analysis population and missing data	#20c	Definition of analysis population relating to protocol non-adherence (eg, as randomised analysis), and any statistical methods to handle missing data (eg, multiple imputation)	17-19
**Methods: Monitoring**			
Data monitoring: formal committee	#21a	Composition of data monitoring committee (DMC); summary of its role and reporting structure; statement of whether it is independent from the sponsor and competing interests; and reference to where further details about its charter can be found, if not in the protocol. Alternatively, an explanation of why a DMC is not needed	24
Data monitoring:	#21b	Description of any interim analyses and stopping	17

For peer review only

interim analysis		guidelines, including who will have access to these interim results and make the final decision to terminate the trial	
Harms	#22	Plans for collecting, assessing, reporting, and managing solicited and spontaneously reported adverse events and other unintended effects of trial interventions or trial conduct	19, 20
Auditing	#23	Frequency and procedures for auditing trial conduct, if any, and whether the process will be independent from investigators and the sponsor	15
**Ethics and dissemination**			
Research ethics approval	#24	Plans for seeking research ethics committee / institutional review board (REC / IRB) approval	25
Protocol amendments	#25	Plans for communicating important protocol modifications (eg, changes to eligibility criteria, outcomes, analyses) to relevant parties (eg, investigators, REC / IRBs, trial participants, trial registries, journals, regulators)	23
Consent or assent	#26a	Who will obtain informed consent or assent from potential trial participants or authorised surrogates, and how (see Item 32)	14
Consent or assent: ancillary studies	#26b	Additional consent provisions for collection and use of participant data and biological specimens in ancillary studies, if applicable	n/a
Confidentiality	#27	How personal information about potential and enrolled participants will be collected, shared, and maintained in order to protect confidentiality before, during, and after the trial	16
Declaration of interests	#28	Financial and other competing interests for principal investigators for the overall trial and each study site	26
Data access	#29	Statement of who will have access to the final trial dataset, and disclosure of contractual agreements that	25, 26

For peer review only

		limit such access for investigators	
Ancillary and post trial care	#30	Provisions, if any, for ancillary and post-trial care, and for compensation to those who suffer harm from trial participation	Not included
Dissemination policy: trial results	#31a	Plans for investigators and sponsor to communicate trial results to participants, healthcare professionals, the public, and other relevant groups (eg, via publication, reporting in results databases, or other data sharing arrangements), including any publication restrictions	25
Dissemination policy: authorship	#31b	Authorship eligibility guidelines and any intended use of professional writers	26-28
Dissemination policy: reproducible research	#31c	Plans, if any, for granting public access to the full protocol, participant-level dataset, and statistical code	25
**Appendices**			
Informed consent materials	#32	Model consent form and other related documentation given to participants and authorised surrogates	Available on request
Biological specimens	#33	Plans for collection, laboratory evaluation, and storage of biological specimens for genetic or molecular analysis in the current trial and for future use in ancillary studies, if applicable	n/a, no specimens

None The SPIRIT checklist is distributed under the terms of the Creative Commons Attribution License CC-BY-ND 3.0. This checklist can be completed online using https://www.goodreports.org/, a tool made by the EQUATOR Network in collaboration with Penelope.ai
